# Direct Targeting of CREB1 with Imperatorin Inhibits TGF*β*2‐ERK Signaling to Suppress Esophageal Cancer Metastasis

**DOI:** 10.1002/advs.202000925

**Published:** 2020-07-01

**Authors:** Wen Wen Xu, Zhi‐Hao Huang, Long Liao, Qi‐Hua Zhang, Jun‐Qi Li, Can‐Can Zheng, Yan He, Ting‐Ting Luo, Yang Wang, Hui‐Fang Hu, Qian Zuo, Wen‐You Chen, Qing‐Sheng Yang, Jian‐Fu Zhao, Yan‐Ru Qin, Li‐Yan Xu, En‐Min Li, Hua‐Xin Liao, Bin Li, Qing‐Yu He

**Affiliations:** ^1^ MOE Key Laboratory of Tumor Molecular Biology and Guangdong Provincial Key Laboratory of Bioengineering Medicine National Engineering Research Center of Genetic Medicine Institute of Biomedicine College of Life Science and Technology Jinan University Guangzhou 510632 China; ^2^ MOE Key Laboratory of Tumor Molecular Biology and Key Laboratory of Functional Protein Research of Guangdong Higher Education Institutes Institute of Life and Health Engineering College of Life Science and Technology Jinan University Guangzhou 510632 China; ^3^ Department of Thoracic Surgery First Affiliated Hospital Jinan University Guangzhou 510632 China; ^4^ Department of Clinical Oncology First Affiliated Hospital Jinan University Guangzhou 510632 China; ^5^ State Key Laboratory of Esophageal Cancer Prevention and Treatment Department of Clinical Oncology First Affiliated Hospital Zhengzhou University Zhengzhou China; ^6^ The Key Laboratory of Molecular Biology for High Cancer Incidence Coastal Chaoshan Area Shantou University Medical College 22 Xinling Road Shantou Guangdong China

**Keywords:** diagnosis, esophageal cancer metastasis, Imperatorin, prognosis, TGF*β*2, therapeutic implications

## Abstract

Metastasis accounts for 90% of cancer death worldwide, and effective therapeutic strategies are lacking. The aim of this work is to identify the key drivers in tumor metastasis and screen therapeutics for treatment of esophageal squamous cell carcinoma (ESCC). Gene Ontology analysis of The Cancer Genome Atlas (TCGA) gene expression datasets of ESCC patients with or without lympy metastasis identifies that TGF*β*2 is highly enriched in the pathways essential for tumor metastasis and upregulates in the metastatic ESCC tumors. High TGF*β*2 expression in ESCC correlates with metastasis and patient survival, and functionally contributes to tumor metastasis via activating extracellular signal‐regulated kinases (ERK) signaling. By screening of a library consisting of 429 bioactive compounds, imperatorin is verified as a novel TGF*β*2 inhibitor, with robustly suppressive effect on tumor metastasis in multiple mice models. Mechanistically, direct binding of imperatorin and CREB1 inhibits phosphorylation, nuclear translocation of CREB1, and its interaction with TGF*β*2 promoter, represses TGF*β*2 expression and fibroblasts‐secreted CCL2, and then inactivates ERK signaling to block cancer invasion and abrogates the paracrine effects of fibroblasts on tumor angiogenesis and metastasis. Overall, the findings suggest the use of TGF*β*2 as a diagnostic and prognostic biomarker and therapeutic target in ESCC, and supports the potential of imperatorin as a novel therapeutic strategy for cancer metastasis.

## Graphical abstract

This work suggests the clinical and functional role of TGF*β*2 in esophageal cancer metastasis. Imperatorin is verified with a robustly suppressive effect on tumor invasion and metastasis in vitro and in vivo. Mechanistically, imperatorin can directly bind with CREB1 and inhibits its phosphorylation and interaction with TGF*β*2 promoter, then inactivates extracellular signal‐regulated kinases (ERK) signaling and fibroblasts‐secreted CCL2 to block tumor angiogenesis and metastasis.

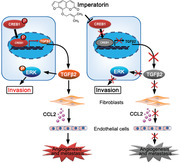

## 1 Introduction

Metastasis is the major cause of treatment failure in cancer patients,^[^ ^1^ ^]^ which involve many key signaling pathways and the communications among multiple cell types.^[^ ^2^ ^]^ The underlying mechanisms in cancer metastasis are largely unknown and the new treatment strategies with good efficacy and safety are lacking. Esophageal cancer ranks as the eighth most common malignancy and sixth most frequent cause of cancer death worldwide, with a survival rate lower than 20%,^[^ ^3^ ^]^ and esophageal squamous cell carcinoma (ESCC) is the predominant type throughout the Asia‐Pacific region including China.^[^ ^4^ ^]^ The high mortality of ESCC is mainly due to late diagnosis and metastasis.^[^ ^5^ ^]^ Despite the recent advances in cancer therapy, the treatment outcome is still not satisfactory. Therefore, there is an urgent need to illustrate the molecular mechanisms in ESCC tumorigenesis and metastasis for development of useful anticancer drugs.

Transforming growth factor beta (TGF*β*) superfamily is a large and continuously expanded group of over 40 multifunctional cytokines, including a model TGF*β* family and other families, such as bone morphogenetic proteins (BMPs), growth and differentiation factors (GDFs). TGF*β* family consists of three isoforms, TGF*β*1, TGF*β*2, and TGF*β*3, and plays an important role in the cell growth and death, differentiation, immune response, angiogenesis and inflammation.^[^ ^6^, ^7^ ^]^ Although efforts have been made to determine the pathological relevance of TGF*β* family, the mechanistic basis and clinical significance of TGF*β* in ESCC remains to be elucidated. In particular, most studies focused on TGF*β*1, the study of TGF*β*2 lags behind. The cAMP‐responsive element‐binding protein 1 (CREB1) is a master nuclear transcription factor activated by multiple extracellular signals, and functions in diverse physiological processes, including cellular metabolism and survival.^[^ ^8^ ^]^ CREB1 binds to the TGF*β*2 gene promoter in cooperation with SMAD3 and is essential for transcriptional activation of TGF*β*2.^[^ ^9^ ^]^ CREB1 has been suggested to be a suitable and attractive cancer target and pharmacological inhibition of CREB1 is a viable strategy for cancer therapy.^[^ ^10^ ^]^ However, only several inhibitors have been developed and none of these inhibitors got satisfactory results or the entry to clinical test.^[^ ^11^ ^]^

A small molecule library consisting of 429 bioactive compounds was taken to screen the drug candidates capable of blocking TGF*β*2 pathway and suppressing tumor invasion, simultaneously, in ESCC. Imperatorin, a bioactive ingredient extracted from the root of angelica dahurica, became our research focus.^[^ ^12^ ^]^ Imperatorin has been reported to have traditional curative effects including anti‐inflammatory, anti‐coagulant, and anti‐bacterial.^[^ ^13^, ^14^, ^15^ ^]^ However, it is still unknown whether imperatorin can inhibit tumor invasion and metastasis. In the present study, imperatorin was found to repress TGF*β*2 expression and invasion in ESCC cells. By performing gain‐ and loss‐of‐function experiments and a series of functional assays, we examined whether imperatorin inhibit TGF*β*2 transcription by directly binding to CREB1. The functional role of TGF*β*2 and therapeutic implication of imperatorin in tumor metastasis and microenvironment were investigated in vitro and in vivo. Our study collectively provides evidence on the functional and clinical significance of TGF*β*2 and the potential of imperatorin as novel therapeutic strategy for cancer treatment.

## 2 Results

### 2.1 Analysis of TCGA Database Suggests that TGF*β*2 is Highly Involved in ESCC Metastasis

**Figure 1:**
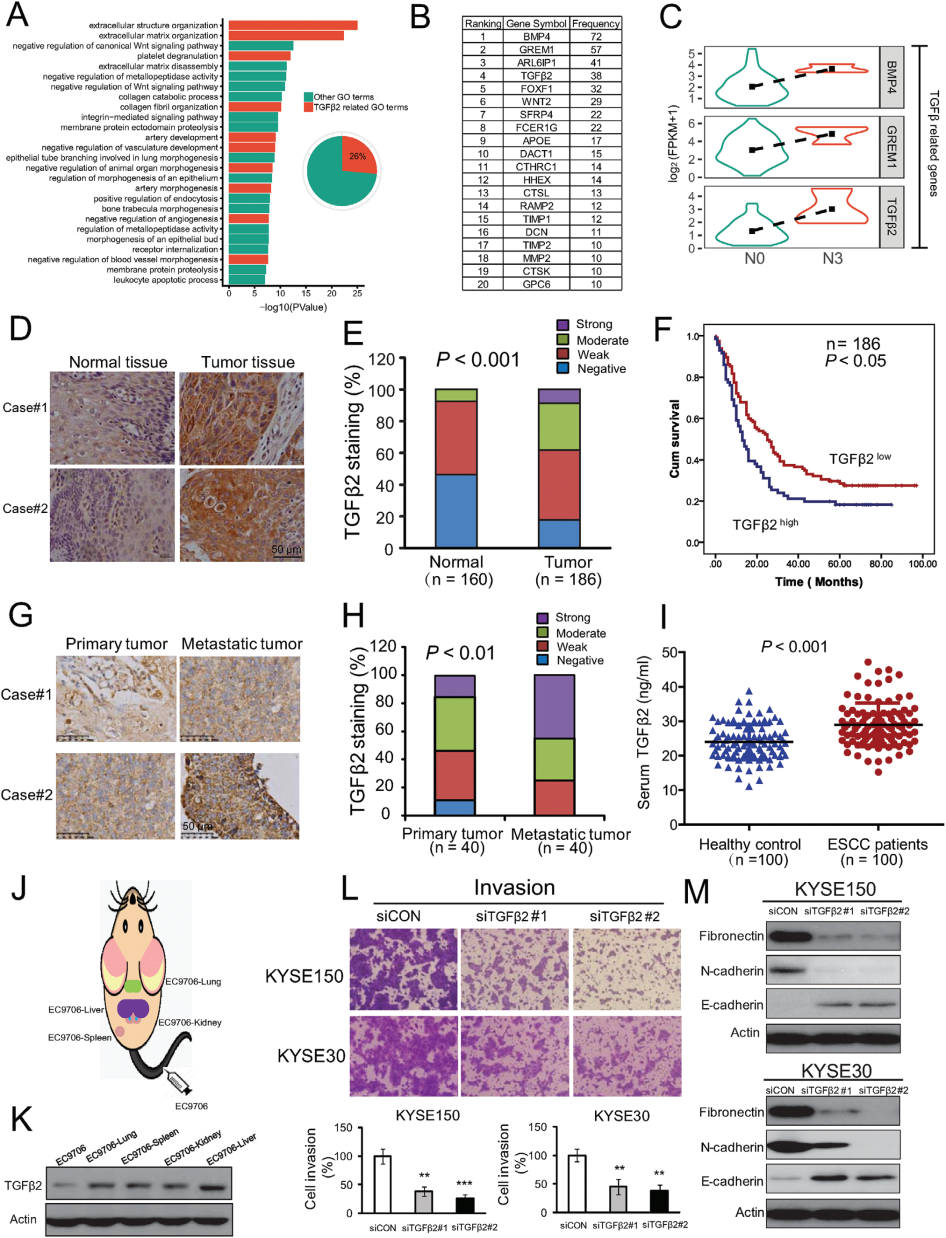
The clinical and functional relevance of TGF*β*2 in ESCC metastasis. A) GO analysis of TCGA gene expression datasets of ESCC patients with or without lympy metastasis. B) A list of the genes most frequently enriched in the GO terms identified in ESCC metastasis. C) TGF*β*2 had a significantly higher expression in N3 tumors than N0 tumors. D) Representative images and E) expression pattern of TGF*β*2 in 186 ESCC and 160 cases of paired normal tissue. F) Kaplan–Meier analysis of overall survival of 186 ESCC patients stratified according to tumor TGF*β*2 expression. G,H) Representative images and expression pattern of TGF*β*2 in 40 primary tumor and matched metastatic tumor. I) Expression of TGF*β*2 in the serum of 100 ESCC patients and 100 healthy control. J) Diagrams showing the mice model and establishment of organ‐specific metastatic cell sublines. K) Expression of TGF*β*2 in parental cell line and organ‐specific metastatic cell sublines. L,M) KYSE150 and KYSE30 cells were transfected with two different siRNAs against TGF*β*2 (50 × 10^−9^ m) for 24 h, and L) cell invasion ability and M) expression levels of EMT markers, were determined by Boyden chamber invasion assay and Western blot, respectively. Bars, SD; **, *P* < 0.01; ***, *P* < 0.001.

To figure out the underlying mechanisms involved in ESCC invasion and metastasis, RNA‐sequencing data and clinical information of 60 ESCC cases were obtained from The Cancer Genome Atlas (TCGA) database, and the gene profiles of tumors at N0 stage were compared with those at N3 stage. Gene Ontology (GO) analysis of the differentially expressed genes indicated that a series of signaling pathways were involved in ESCC metastasis, such as extracellular matrix organization (**Figure** **1**A). The frequency of each gene to be enriched in the pathways was calculated to identify the key drivers which affect most of the important signaling pathways involved in cancer invasion and metastasis. Among the top genes highly enriched in the pathways listed in Figure 1A, TGF*β*2, which ranks 4th with a high frequency (26%) of enrichment in the total GO terms, became our research focus (Figure 1B). As shown in Figure 1C, TGF*β*2 had a significantly higher expression in the ESCC tumors with high metastasis compared with the tumors without metastasis (Figure 1C). These analyses of TCGA data suggest that TGF*β*2 may play a crucial role in metastasis of esophagus cancer.

### 2.2 Clinical Significance of TGF*β*2 Expression in ESCC

The expression level and clinical relevance of TGF*β*2 in esophageal cancer remain largely unknown. Firstly, we determined TGF*β*2 expression in 186 cases of primary ESCC tumor tissues and 160 cases of matched adjacent normal tissues by immunohistochemistry (Figure S1A, Supporting Information). The majority of the tumor tissues had stronger TGF*β*2 immunostaining than corresponding normal tissues. As shown in Figure 1D,E, 38.17% (71/186) of tumor tissues displayed high TGF*β*2 expression, but only 7.50% (12/160) of normal tissues had high TGF*β*2 expression. In contrast, 92.50% (148/160) of normal tissues, whereas only 61.83% (115/186) of tumor tissues, showed low TGF*β*2 expression. Kaplan–Meier survival analysis indicated that the patients with high TGF*β*2 expression had significantly shorter survival (median survival = 13.0 months) than the patients with lower tumor TGF*β*2 expression (median survival = 25.0 months) (log‐rank = 5.65, *P* < 0.05, Figure 1F, Table S1, Supporting Information). Moreover, high TGF*β*2 expression was significantly associated with lymph node metastasis (Pearson *χ*2 test, *P* < 0.001; **Table** **1**). Another tissue microarray consisting of 40 primary ESCC tumors and matched metastatic tissues was further used to detect TGF*β*2 expression, and the immunohistochemistry data showed that the majority of the metastatic tumor tissues had higher TGF*β*2 expression than the corresponding primary tumors (Figure 1G,H), corroborating our findings above that high TGF*β*2 expression is correlated with metastasis and poor prognosis. Furthermore, serum TGF*β*2 level in 100 ESCC patients and 100 healthy individuals was compared by using Enzyme‐Linked Immunosorbent Assay (ELISA), and a markedly higher concentration of TGF*β*2 was observed in ESCC patients (Figure 1I). Among the 100 cases ESCC patients, there are 73 cases with metastasis information, and we noted that TGF*β*2 level was higher in the serum of ESCC patients with metastasis than those without metastasis (Figure S1B, Supporting Information). These results collectively suggest that circulation TGF*β*2 may serve as a non‐invasive biomarker in ESCC. We also detected TGF*β*2 expression in 12 pairs of ESCC primary tumor tissues and matched non‐tumor tissues by Western blot, and the results revealed that TGF*β*2 is overexpressed in the majority of ESCC tumors (9 cases/12 cases, 75.0%), compared with matched normal tissues (Figure S1C, Supporting Information). In addition, we analyzed TGF*β*2 expression and its correlation with pathological features by using the gene expression profiling interaction analysis (GEPIA) database,^[^ ^16^ ^]^ and found that TGF*β*2 was significantly upregulated in esophageal carcinoma (ESCA), glioblastoma (GBM) and pancreas adenocarcinoma (PAAD), compared with corresponding normal tissue, respectively (Figure S1D, Supporting Information). TGF*β*2 expression was found to be positively associated with the pathological stages of esophageal cancer (Figure S1E, Supporting Information). Taken together, these data suggest that TGF*β*2 may be potentially useful as a diagnostic and prognostic biomarker for esophageal cancer.

**Table 1:** Correlation between TGF*β*2 expression levels and clinicopathological parameters in 186 cases of esophageal cancer

Variable	*n*	Low TGF*β*2	High TGF*β*2	*P* value^a^^)^
Age (years)				
≤55	29	19	10	
>55	157	96	61	0.656
				
Gender				
Male	144	89	55	
Female	42	26	16	1
				
T‐Stage				
1/2	35	26	9	
3/4	141	81	60	0.067
				
N‐Stage				
N0	86	64	22	
N1/N2/N3	100	51	49	0.001**
				
M‐Stage				
M0	186	115	71	
M1	0	0	0	1
				
Grade				
I & II	144	67	57	
III & IV	42	28	14	0.152
				

a ^)^Pearson Chi‐square. Statistical significance (*P* < 0.05) is shown in bold

### 2.3 TGF*β*2 Promotes ESCC Invasion and Metastasis

The functional role of TGF*β*2 in ESCC metastasis remains to be elucidated. In a multi‐organ metastasis model established by intravenously injecting luciferase‐expressing ESCC cells EC9706‐Luc into the tail vein of NOD‐Prkdc^em26Cd52^Il2rg^em26Cd22^ (NCG) mice, we isolated a series of metastatic cell sublines, designated as EC9706‐lung, EC9706‐kidney, EC9706‐liver, and EC9706‐spleen, which specially metastasized to lung, kidney, liver, and spleen, respectively (Figure 1J). Western blot data showed that TGF*β*2 was significantly upregulated in all of the metastatic cell sublines, compared with parental cells (Figure 1K), suggesting that TGF*β*2 is essential to drive tumor metastasis.

To examine the biological function of TGF*β*2 in cancer motility, loss‐of‐function experiment and Boyden chamber assay was performed, and the results showed that knockdown of TGF*β*2 significantly reduced the invasive potential of KYSE150 and KYSE30 cells (Figure 1L; Figure S1F, Supporting Information). Increased E‐cadherin expression and decreased expression of Fibronectin and N‐cadherin were found in TGF*β*2‐knockdown ESCC cells (Figure 1M). The opposite effects on expression levels of the epithelial‐mesenchymal transition (EMT) markers was observed in the KYSE150 and KYSE30 cells exposed to increasing doses of TGF*β*2 recombinant protein (Figure S1G, Supporting Information). Exogenous TGF*β*2 also enhanced the invasive capacity of ESCC cells in a dose‐dependent manner (Figure S1H, Supporting Information). Meanwhile, the cell viability was monitored and no apparent alternation of cell growth was observed in the TGF*β*2‐treated cells, which excluding the possibility that the anti‐invasive effect of TGF*β*2 was due to its anti‐proliferation ability (Figure S1I, Supporting Information). The results above collectively demonstrated the importance of TGF*β*2 in ESCC metastasis.

### 2.4 Identification of Imperatorin as a Novel Inhibitor of TGF*β*2 and Cancer Invasion

**Figure 2:**
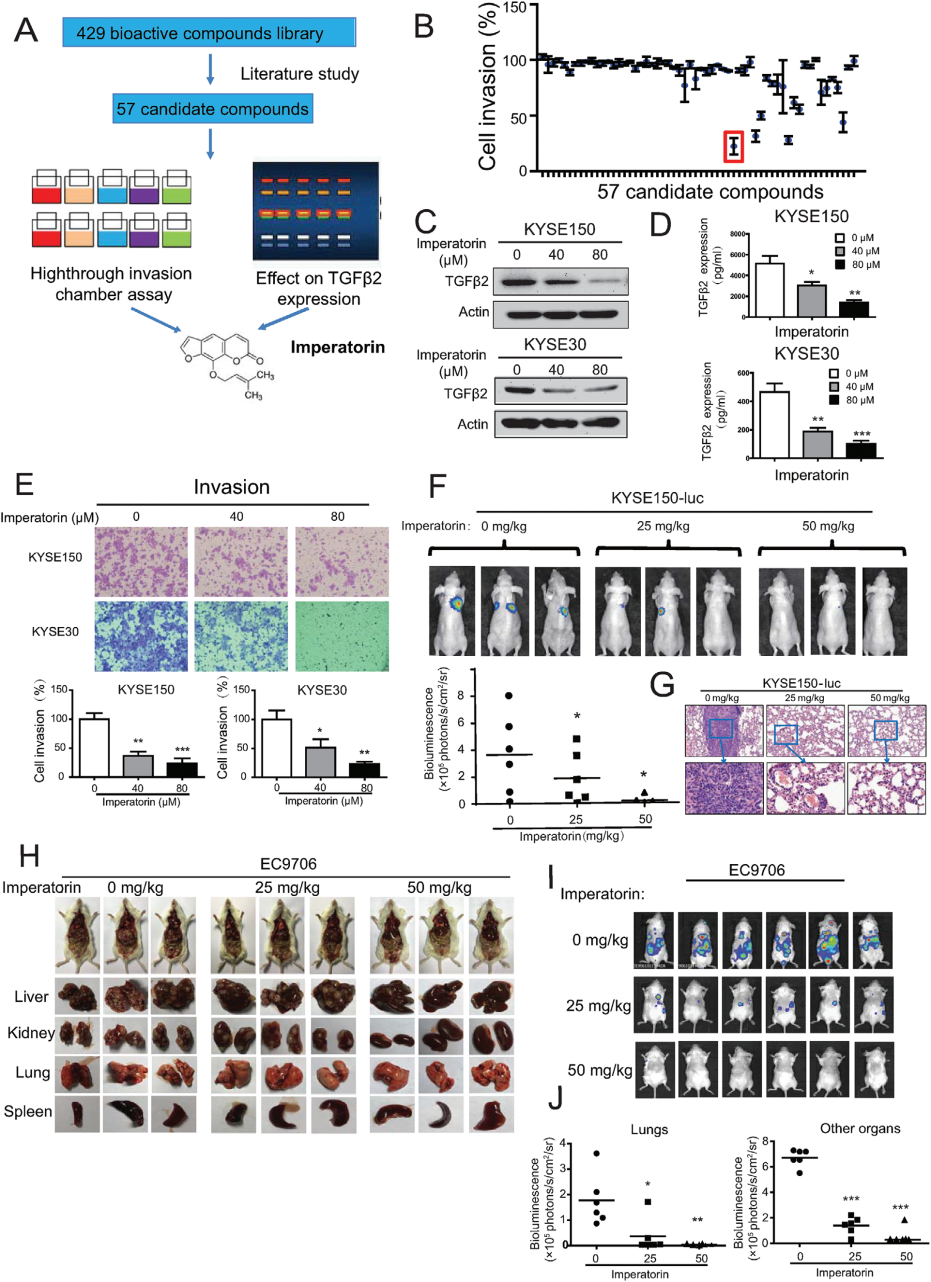
Identification of imperatorin as a novel inhibitor of TGF*β*2 and tumor metastasis. A) A diagram showing the approach to screen imperatorin as a potential inhibitor of TGF*β*2 and tumor invasion. B) Quantification of the anti‐invasive effect of 57 candidate compounds in KYSE150 cells. C–E) KYSE150 and KYSE30 cells were treated with increasing concentration of imperatorin for 24 h, and Western blot was used to detect C) TGF*β*2 expression in cells and D) ELISA assay was performed to determine the secretin level of TGF*β*2 in the conditioned media, and E) chamber invasion assay was used to examine cell invasion. F) Bioluminescence imaging and quantification of lung metastasis in the nude mice that were intravenously injected with KYSE150‐Luc cells and treated with imperatorin (25 and 50 mg kg^−1^) or vehicle. G) Hematoxylin & eosin (H & E) staining showing the lung metastasis. H) Anatomical analysis of the organs with multi‐metastasis in the NCG mice that were intravenously injected with EC9706‐Luc cells and treated with imperatorin (25 and 50 mg kg^−1^) or vehicle. I,J) Bioluminescence imaging and quantification of metastasis in lung, liver, kidney, and spleen . Bars, SD; *, *P* < 0.05; **, *P* < 0.01; ***, *P* < 0.001.

To identify the drug candidates capable of targeting TGF*β*2 and suppressing tumor invasion simultaneously, a small molecule library consisting of 429 bioactive compounds was used for initial screening by literature study. In total, 57 candidate compounds, which have not been reported to exert any effect in cancer invasion, was taken and their effect on TGF*β*2 expression and cell invasion was determined by using Western blot and Boyden chamber assay, respectively, in ESCC cells (**Figure** **2**A; Figure S2A,B and Table S2, Supporting Information). Imperatorin, a naturally occurring furanocoumarin, was the most effective compound in inhibiting both TGF*β*2 expression and cell invasion, became our research focus (Figure 2B,C).

As shown in Figure 2D, imperatorin not only reduced TGF*β*2 expression at protein level, but also led to a dose‐dependent decrease in TGF*β*2 secretin in the conditioned medium. Imperatorin inhibited invasive potential of KYSE150 and KYSE30 cells in a dose‐dependent manner (Figure 2E). We got consistent results in the migration assay (Figure S2C, Supporting Information). The results from cell viability assay showed that imperatorin had no obvious effect on cell proliferation, excluding the possibility that the anti‐invasive effect of imperatorin was due to its anti‐proliferation ability (Figure S2D, Supporting Information). Western blot analysis indicated that treatment with imperatorin increased E‐cadherin expression and decreased expression levels of fibronectin, N‐cadherin, snail, MMP2, and MMP9 (Figure S2E, Supporting Information). Moreover, no obvious cytotoxic effect was found in the normal esophageal epithelial cells treated with imperatorin (Figure S2F, Supporting Information), suggesting that imperatorin may be a potential anticancer agent with good safety.

### 2.5 Imperatorin Suppresses Tumor Metastasis Without Overt Side Effect

Next, the treatment efficacy and potential toxicity of imperatorin were evaluated in animals. Nude mice were intravenously injected with luciferase‐expressing KYSE150 cells (KYSE150‐Luc) through tail vein to establish tumor metastasis, and then orally administrated with different doses of imperatorin (25 or 50 mg kg^−1^). Bioluminescent imaging data showed that treatment with imperatorin led to a significant decrease of lung metastasis in a dose‐dependent manner (Figure 2F), which was also evidenced by histological examination of the lungs (Figure 2G). There was no obvious difference between the treated and control groups in terms of body weight (Figure S3A, Supporting Information) and serum level of ALT and AST (Figure S3B, Supporting Information). Histological analysis did not show observed changes in morphology of liver and kidney (Figure S3C, Supporting Information). Furthermore, the anticancer bioactivity of imperatorin was examined in a multi‐organ metastasis animal model, in which EC9706‐Luc cells were intravenously into the tail vein of NCG mice and the mice were treated with increasing doses of imperatorin (25 or 50 mg kg^−1^). We found that imperatorin not only inhibited the metastatic colonization of ESCC cells to the lungs, but also other organs including liver, kidneys and spleen (Figure 2H–J). Moreover, the imperatorin treatment had no toxic effect on animals, evidenced by unchanged body weight, as well as the histological and hematologic analyses (Figure S3D–F, Supporting Information).

### 2.6 Proteomics Analysis Suggested the Inactivation of TGF*β*2‐extracellular signal‐regulated kinases (ERK) Signaling Induced by Imperatorin

**Figure 3:**
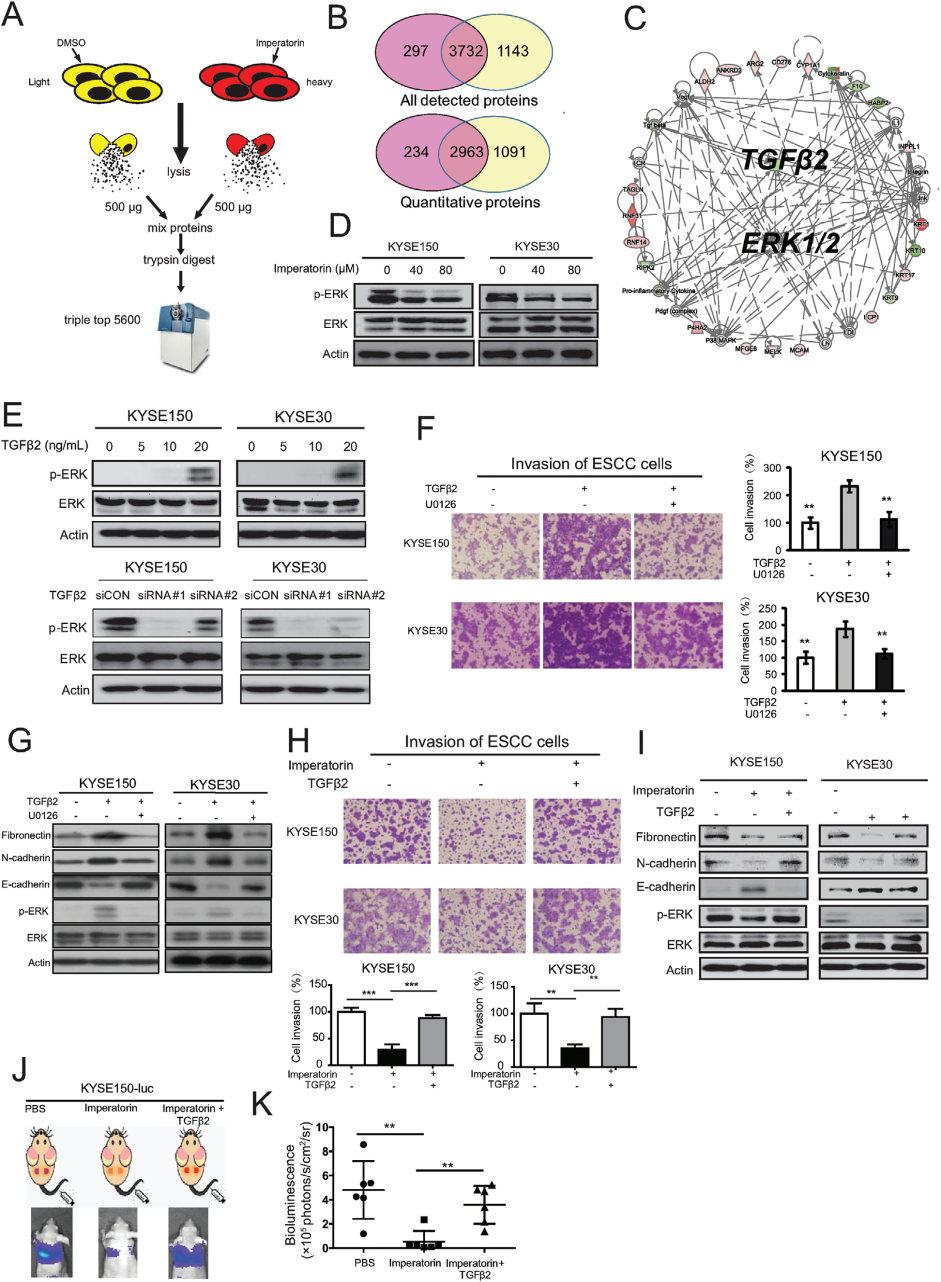
Proteomics analysis suggested the inactivation of TGF*β*2‐ERK signaling induced by imperatorin A) Experimental scheme showing the identification of imperatorin‐regulated proteins by SILAC quantitative proteomics. B) Venn diagram showing the identification of overlapped proteins in two biological replicates. C) Differentially expressed proteins with fold change ≥1.5 were subjected to IPA analysis. D) Western blot analysis of p‐ERK and ERK expression in the KYSE150 and KYSE30 cells treated with imperatorin (up to 80 × 10^−6^ m) for 24 h. E) KYSE150 and KYSE30 cells were exposed to indicated doses of TGF*β*2 recombinant protein, or transfected with the siRNA against TGF*β*2 (50 × 10^−9^ m), respectively, for 24 h, and expression levels of p‐ERK and ERK were determined by Western blot. F,G) KYSE150 and KYSE30 cells were exposed to TGF*β*2 (20 ng mL^−1^) with or without U0126 (10 × 10^−6^ m) for 24 h, and cell invasion was compared by F) Boyden chamber assay and G) expression of Fibronectin, N‐cadherin, E‐cadherin, p‐ERK, and ERK was detected by Western blot. H) Boyden chamber assay comparing the invasive potential of KYSE150 and KYSE30 cells treated with imperatorin (80 × 10^−6^ m) for 24 h in the presence or absence of TGF*β*2. I) TGF*β*2 attenuated the effect of imperatorin on expression of the EMT markers and p‐ERK. J,K) Bioluminescence imaging and quantification of metastasis in lungs as indicated. Bars, SD; **, *P* < 0.01; ***, *P* < 0.001.

To investigate the underlying mechanisms how imperatorin inhibits tumor invasion, Stable Isotope Labeling by Amino Acids in Cell Culture (SILAC)‐based quantitative proteomics were performed to explore the proteins regulated by imperatorin (**Figure** **3**A). A total of 2963 proteins with quantitative information were both identified in the duplicated experiments (Figure 3B), and 127 proteins were found to be differentially expressed (fold change ≥1.5) in the ESCC cells treated with 40 × 10^−6^ m imperatorin for 24 h (Table S3, Supporting Information). As expected, TGF*β*2 expression was found to be significantly downregulated b y imperatorin treatment. Ingenuity pathway analysis (IPA) was used to characterize the canonical pathways that the 127 differentially expressed proteins participated, and TGF*β*2‐ERK signaling was predicted to play a hub role in the action mechanism of imperatorin (Figure 3C). We checked the expression of p‐ERK and ERK in the metastatic cell lines and found the activation of ERK signaling, compared with parental cell line (Figure S4A, Supporting Information). This hypothesis was confirmed by our Western blot results showing that ERK signaling was significantly inactivated in imperatorin‐treated KYSE150 and KYSE30 cells (Figure 3D). The positive regulation of ERK signaling by TGF*β*2 was demonstrated by addition of TGF*β*2 recombinant protein, and reversely, inhibition of TGF*β*2 with siRNA markedly decreased p‐ERK expression in KYSE150 and KYSE30 cells (Figure 3E). Our results showed that blockade of MEK pathway with U0126 significantly abrogated the promoting effect of TGF*β*2 on cancer cell invasion and EMT phenotype (Figure 3F,G). Moreover, addition of exogenous TGF*β*2 abolished the inhibitory effect of imperatorin on cancer cell invasion and EMT, suggesting that TGF*β*2 mediates the effect of imperatorin on cancer invasion (Figure 3H,I). The in vivo rescue experiment was established and the data showed that treatment with imperatorin resulted in the delay of tumor metastasis, whereas intravenous injection of TGF*β*2 significantly abolished this effect (Figure 3J,K). We further determined the expression of TGF*β*2 and p‐ERK in the ESCC tumor xenografts and as shown in Figure S4B in the Supporting Information, the treatment of imperatorin led to the decrease of TGF*β*2 expression and inactivation of ERK signaling in vivo. Taken together, these data indicated that imperatorin inactivates TGF*β*2‐ERK signaling to inhibit cancer invasion.

### 2.7 Imperatorin Suppresses Tumor Angiogenesis through Inhibition of Cancer‐Associated Fibroblasts (CAFs)‐Secreted CCL2

**Figure 4:**
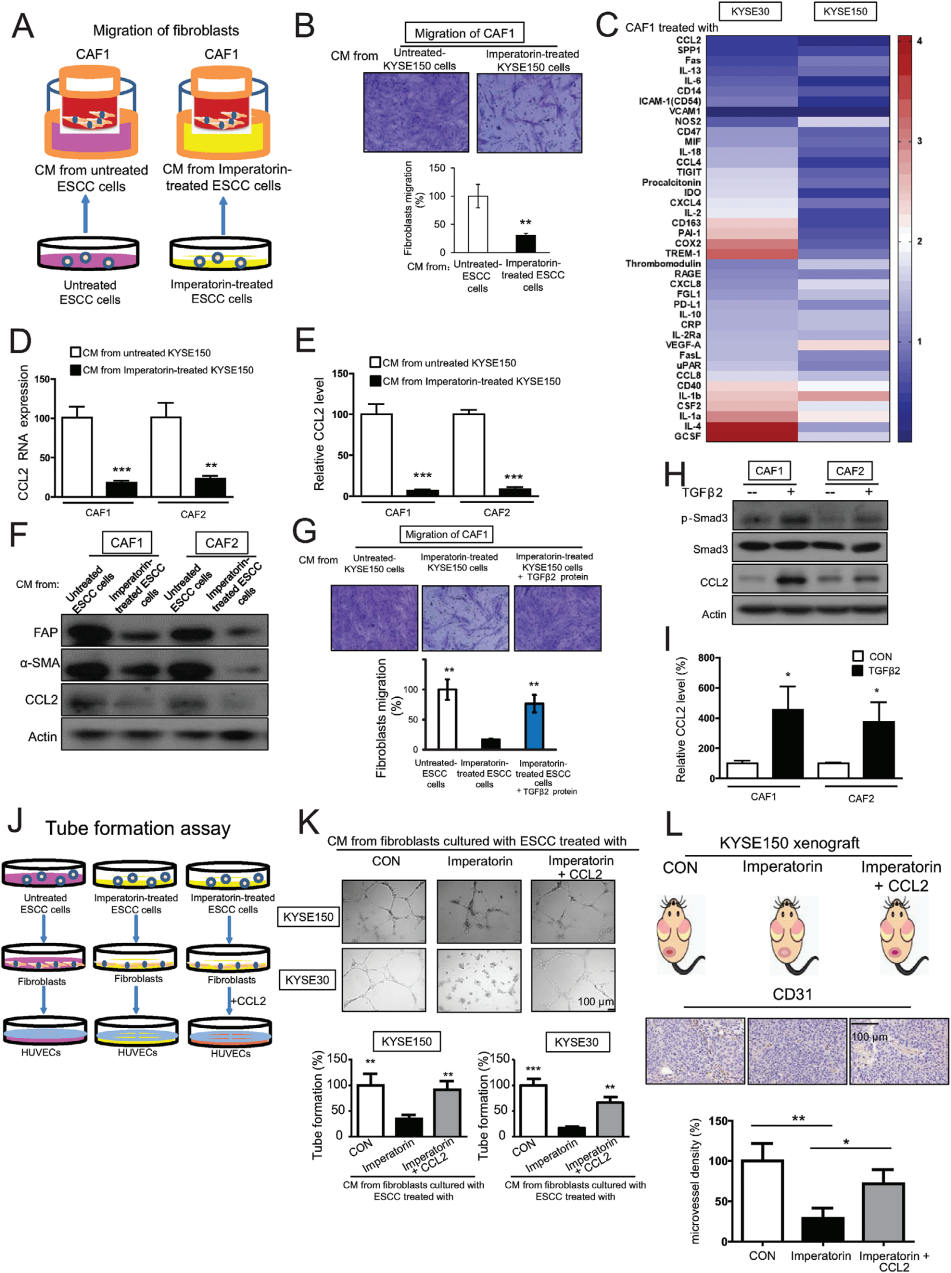
Influence of imperatorin in tumor microenvironment. A) Diagram showing the approach to collect the conditioned medium (CM) from imperatorin‐treated ESCC cells or untreated ESCC cells to attract the migration of cancer‐associated fibroblasts (CAFs). B) Migration assay showing the migration of CAFs attracted by CM from the KYSE150 cells exposed to imperatorin (80 × 10^−6^ m) for 24 h, or the untreated KYSE150 cells, respectively. C) The CM from the KYSE30 and KYSE150 cells treated with imperatorin (80 × 10^−6^ m) for 24 h, and the untreated cells, were collected, respectively, to culture CAFs. The mRNA expression of immune‐related genes were examined by qRT‐PCR and the fold change was presented in heat map. D–F) qRT‐PCR, ELISA, and Western blot were used to detect the D) RNA, E) secretion, and F) protein level of CCL2, as well as expression of FAP and *α*‐SMA in CAF1 and CAF2 when treated with the CM from imperatorin‐treated or untreated ESCC cells, respectively. G) Migration assay showing the cell migration of CAFs attracted by CM from imperatorin‐treated ESCC cells in presence or absence of TGF*β*2. H) Western blot analysis of p‐Smad3, Smad3, and CCL2 expression in the CAFs exposed to TGF*β*2 for 24 h. I) qRT‐PCR was taken to determine the CCL2 expression at the RNA level. J) Diagram showing the approach of collecting CM from imperatorin‐treated or untreated ESCC cell to study the paracrine effect of CAFs on HUVECs. K) Tube formation assay showing the in vitro angiogenesis of HUVECs cultured in the indicated different CM from CAFs with or without addition of CCL2 (5 ng mL^−1^). L) Diagram showing the establishment of tumor xenografts and quantification of MVD, indicated by CD31. Bars, SD; *, *P* < 0.05; **, *P* < 0.01; ***, *P* < 0.001.

We further investigated whether imperatorin can influence the paracrine effect of cancer cells on various stromal cells in tumor microenvironment. The conditioned media (CM) from imperatorin‐treated or untreated ESCC cells were collected as chemoattractant to attract the migration of CAFs (**Figure** **4**A), and a significant decrease in migration of CAFs was observed when the ESCC cells were exposed to imperatorin (Figure 4B). To illustrate the mechanisms involved, CAFs were exposed to different CM from imperatorin‐treated or untreated ESCC cells, respectively, and compared by quantitative real‐time polymerase chain reaction (qRT‐PCR) for expression levels of a series of immune related cytokines such as vascular endothelial growth factor (VEGF), interleukin‐6 (IL‐6), fibroblasts‐secreted C‐C motif chemokine ligand 8 (CCL8). Note that CCL2 was the most downregulated cytokine in the CAFs exposed to CM from imperatorin‐treated ESCC cells (Figure 4C). A dose‐dependent decrease of CCL2 in CAFs upon cultured with CM from imperatorin‐treated ESCC cells was confirmed at RNA, protein and secretion level by qRT‐PCR, Western blot and ELISA, respectively (Figure 4D–F). In addition, we found that the CAFs not only had markedly reduced expression of CAFs markers, including *α*‐SMA and FAP, but also exhibited significantly weaker migrative potential, when they were cultured with the CM from imperatorin‐treated ESCC cells compared with those exposed to control CM (Figure 4F,G), and this could be rescued when TGF*β*2 recombination protein were added to the CM derived from imperatorin‐treated ESCC cells. The direct involvement of TGF*β*2 in these effects was further confirmed by the data showing that TGF*β*2 induced CCL2 expression in CAFs through activation of Smad3 signaling pathways (Figure 4H), which has been reported as a transcription factor of CCL2.^[^ ^17^ ^]^ The addition of TGF*β*2 led to increased CCL2 expression at both protein and RNA levels (Figure 4I). Moreover, the CM from imperatorin‐treated ESCC cells were found to reduce the paracrine effect of CAFs on tumor microenvironment by attenuating the in vitro angiogenic activity of HUVECs and invasion of cancer cells induced by CAFs, compared with the CM from untreated ESCC cells, whereas the addition of CCL2 recombinant protein restored the effect (Figure 4J,K; Figure S4C,D, Supporting Information). Furthermore, microvessel density (MVD) was determined by immunohistochemical staining of CD31 in the subcutaneous ESCC tumor xenografts treated with imperatorin or vehicle control, and our results showed that treatment with imperatorin resulted in a significant decrease of MVD, whereas systemic injection of CCL2 via tail vein abolished the suppressive effect of imperatorin on tumor angiogenesis (Figure 4L). We further established the in vivo experiments to determine the paracrine effects of fibroblasts on tumor metastasis. The mice were intravenously injected with KYSE150‐Luc cells and treated with imperatorin in presence or absence of CCL2. The data indicated that imperatorin suppressed metastasis of ESCC cells, whereas the injection of CCL2 via tail vein, which mimicked a “rescue” paracrine effects of fibroblasts, significantly attenuated the inhibitory effect of imperatorin on tumor metastasis (Figure S4E,F, Supporting Information).

Collectively, these results provide evidence showing that imperatorin may inhibit TGF*β*2 expression in cancer cells to suppress CAFs‐secreted CCL2 and the subsequent paracrine effect on endothelial cells and cancer cells, therefore, blocking tumor angiogenesis and invasion.

### 2.8 Imperatorin Directly Targets CREB1 and Inhibits TGF*β*2 Transcription

**Figure 5:**
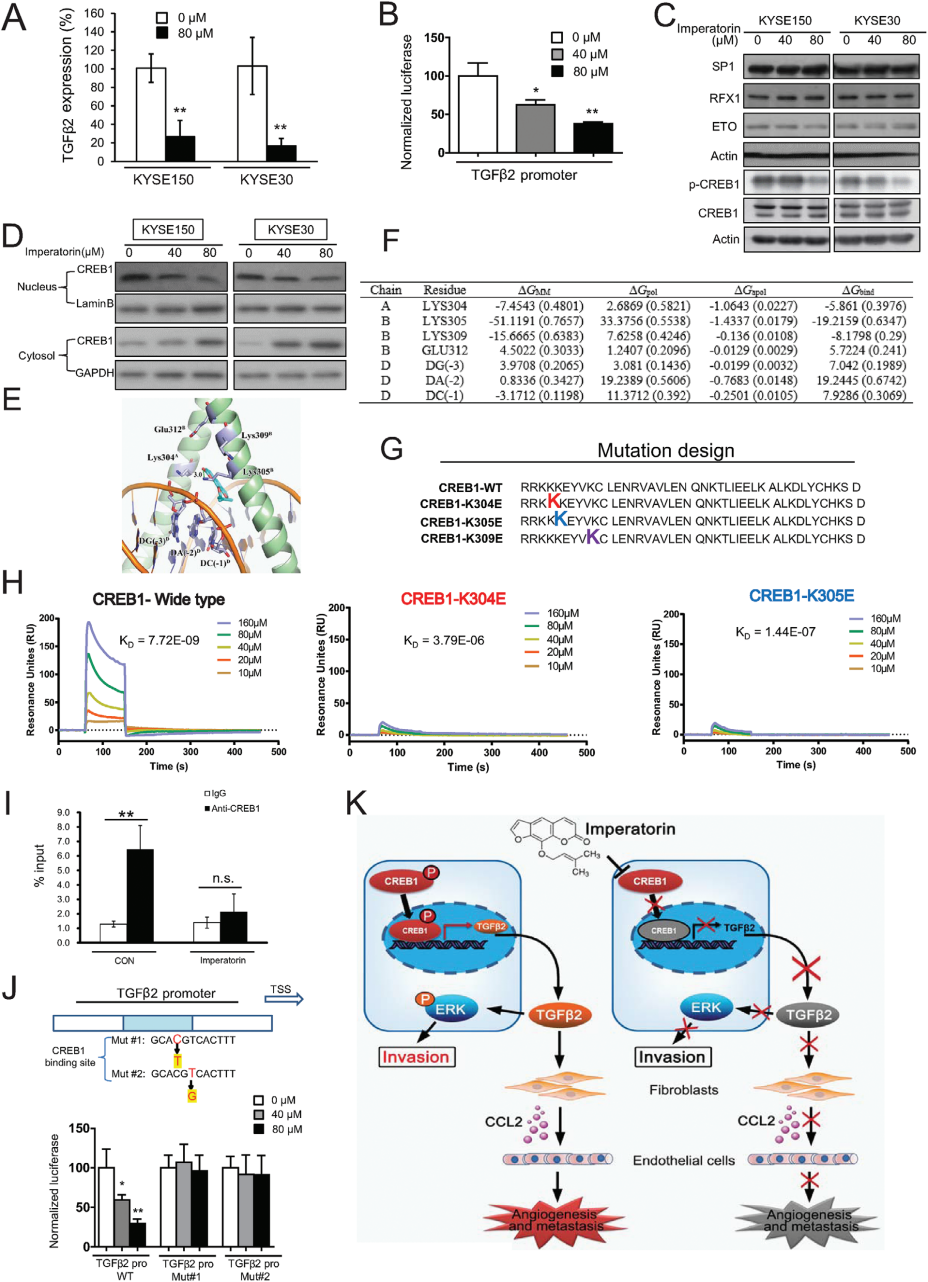
Imperatorin directly binds to CREB1 and disrupts the interaction between CREB1 and TGF*β*2 promoter. A) KYSE150 and KYSE30 cells were treated with imperatorin (80 × 10^−6^ m) for 24 h, and mRNA expression of TGF*β*2 was determined by qRT‐PCR. B) The luciferase assay showing the effect of imperatorin on luciferase activity of TGF*β*2 promoter in KYSE150 cells. C) Western blot data showed that imperatorin decreased p‐CREB1 expression, but not the expression levels of CREB1, SP1, RFX1, and ETO. D) KYSE150 and KYSE30 cells were treated with indicated concentrations of imperatorin for 24 h, and Western blot were performed to detect CREB1 expression in the nucleus and cytoplasm. E) The homology modeling structure of CREB1 protein complexed with DNA. F) Key residues and their energetic contributions identified by the MD simulation and MM/PBSA calculation. G) Diagram showing the mutation design. H) BIAcore analysis revealing the binding between imperatorin and CREB1 protein. I) The enrichment of CREB1 in TGF*β*2 promoter and its inhibition by imperatorin treatment was examined by ChIP‐qPCR. J) The diagram illustrating the mutation design of TGF*β*2 promoter (Upper panel). Lower panel showed the luciferase activity in KYSE150 cells transfected with the plasmid expressing TGF*β*2‐promoter WT, TGF*β*2‐promoter Mut#1, or TGF*β*2‐promoter Mut#2, respectively, in presence or absence of indicated concentrations of imperatorin. K) Schematic diagram summarizing how imperatorin exerts intracellular and extracellular effects to suppress tumor invasion and metastasis. Bars, SD; *, *P* < 0.05; **, *P* < 0.01.

To investigate the molecular mechanisms how imperatorin regulates TGF*β*2 expression, we examined whether imperatorin could affect TGF*β*2 transcription, and the results from qRT‐PCR experiment showed that imperatorin significantly reduced TGF*β*2 expression at mRNA level in KYSE150 and KYSE30 cells (**Figure** **5**A). Next, the promoter region of TGF*β*2 was cloned into pGL3 backbone to generate the plasmid pGL3‐TGF*β*2‐pro, and a dose‐dependent decrease in transcriptional activity of TGF*β*2 promoter was observed in imperatorin‐treated cells, indicated by dual‐luciferase reporter assay (Figure 5B). These findings led us to propose that imperatorin may interact with the transcription factors of TGF*β*2, thus negatively regulating its transcription. KYSE150 and KYSE30 cells were treated with increasing concentrations of imperatorin, and Western blot was performed to determine expression levels of specificity protein 1 (SP1),^[^ ^18^ ^]^ Regulatory Factor X1 (RFX1),^[^ ^19^ ^]^ Eight twenty one protein (ETO)^[^ ^20^ ^]^ and CREB1,^[^ ^9^ ^]^ the transcription factors reported to regulate TGF*β*2 transcription. None of the transcription factors was affected by imperatorin, but interestingly, a dose‐dependent decrease of p‐CREB1 expression was observed in imperatorin‐treated ESCC cells (Figure 5C). More importantly, nuclear translocation of CREB1, which was required for its functional role as transcription factor, was markedly repressed upon exposure to imperatorin in KYSE150 and KYSE30 cells, suggesting the crucial role of CREB1 in the regulation of TGF*β*2 transcription by imperatorin (Figure 5D).

Next, we examined whether imperatorin directly target CREB1 by bioinformatics analysis and chemical biology technology. Since no crystal structure of CREB1 protein was reported yet, the homology modelling was performed to predict the 3D structure of CREB1 protein. As shown in Figure 5E, the structure of CREB1 was built by SWISS‐MODEL software (http://swissmodel.expasy.org). The possible binding pockets were determined by online software POCASA 1.1 (http://altair.sci.hokudai.ac.jp/g6/service/pocasa). Molecular docking and dynamics simulation showed that imperatorin may interact with CREB1 protein mostly likely through hydrophobic interaction with Lys‐304 and Lys‐309 as well as hydrogen bonding with Lys‐305, according to the desolvation energies (Figure 5F). To confirm the computational prediction, we purified CREB1 protein, as well as the CREB1^K304E^ and CREB1^K305E^ proteins, in which Lys‐304 and Lys‐305 was mutated, but failed to purify the CREB1^K309E^ protein (Figure 5G). The results from surface plasmon resonance (SPR) assay showed that imperatorin can bind to CREB1 with a *K*_d_ of 7.72 × 10^−9^, which was disrupted when Lys‐304 or Lys‐305 of CREB1 was mutated (*K*_d_^K304E^ = 3.79 × 10^−6^, *K*_d_^K305E^ = 1.44 × 10^−7^) (Figure 5H). These findings provided direct evidence that imperatorin preferentially binds to the wide‐type CREB1 protein rather than the mutants, suggesting the crucial role of Lys‐304 and Lys‐305 in this process.

To detect the biological effect of the interaction between imperatorin and CREB1, chromatin immunoprecipitation (ChIP) assay was used to evaluate the binding of CREB1 to promoter region of TGF*β*2. As shown in Figure 5I, the interaction between CREB1 and TGF*β*2 promoter was markedly inhibited by imperatorin. We next examined the effect of imperatorin on transcriptional activity of TGF*β*2 promoter. The data from dual‐luciferase reporter assay indicated that imperatorin significantly reduced luciferase activity of TGF*β*2 promoter, and this effect was diminished when the binding sites of CREB1 on the TGF*β*2 promoter were mutated (Figure 5J). Taken together, these data demonstrated that imperatorin directly targets and inhibits the binding of CREB1 protein to TGF*β*2 promoter, and then suppresses TGF*β*2 transcription and expression.

### 2.9 Clinical Relevance of p‐CREB1 in ESCC

**Figure 6:**
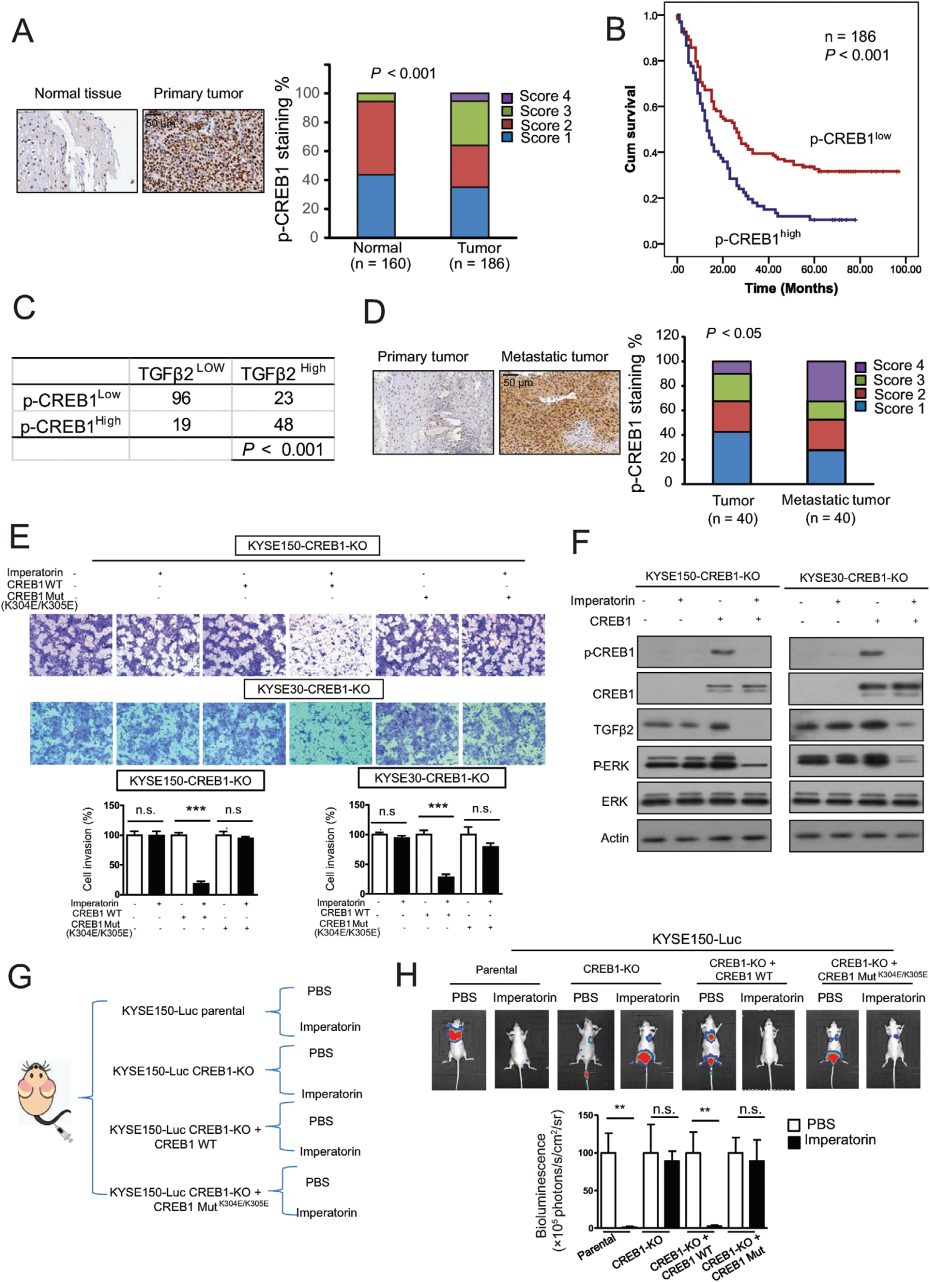
CREB1 is essential for the suppressive effect of imperatorin on TGF*β*2‐ERK signaling and ESCC metastasis. A) Representative images and expression pattern of p‐CREB1 in 186 ESCC and 160 cases paired normal tissues. B) Kaplan–Meier analysis of overall survival of 186 ESCC patients stratified according to tumor p‐CREB1 expression. C) The p‐CREB1 expression was positively associated with TGF*β*2 expression in ESCC patients. D) Representative images and expression pattern of p‐CREB1 in 40 primary ESCC and matched metastatic tumors. E,F) KYSE150‐CREB1‐KO and KYSE30‐CREB1‐KO cells were further overexpressed with wild‐type CREB1, mutant CREB1, or vector control. E) Effect of imperatorin on invasion of these cells was compared by Boyden chamber assay. F) Western blot was used to determine TGF*β*2 and p‐ERK expression. G) Experimental scheme illustrating the animal experiment design. H) Bioluminescence imaging and quantification showed that imperatorin could not suppress the ability of CREB1‐knockout KYSE150‐Luc cells to form tumor metastasis, and the anti‐metastatic effect of imperatorin was recovered when the cells were re‐overexpressed with wild‐type CREB1, but not the mutant CREB1. Bars, SD; **, *P* < 0.01; ***, *P* < 0.001.

The clinical significance of p‐CREB1 expression in ESCC remains unknown. Immunohistochemistry was performed to determine p‐CREB1 expression in the 186 primary tumors and matched 160 non‐tumor tissues (Figure S5A, Supporting Information). We found that the expression of p‐CREB1 was markedly higher in the tumors than the paired normal tissues (*P* < 0.001) (**Figure** **6**A), moreover, p‐CREB1 expression was significantly associated with lymph node metastasis (Pearson *χ* ^2^ test, *P* = 0.014) (Table S4, Supporting Information). Kaplan–Meier survival analysis showed that the patients with high p‐CREB1 expression in their tumors had significantly shorter survival (median survival = 13.0 months) than the patients with low tumor p‐CREB1 expression (median survival = 26.0 months) (log‐rank = 13.28, *P* < 0.001, Figure 6B). More importantly, a strong positive correlation between expression of TGF*β*2 and p‐CREB1 was observed (Pearson *χ* ^2^ test, *P* < 0.001) (Figure 6C), which corroborated our findings above on the important role of CREB1 in regulation of TGF*β*2 expression. Furthermore, p‐CREB1 expression was detected in another tissue microarray containing 40 pairs of primary tumors and metastatic tumor tissues, and the result showed that majority of the metastatic tumors displayed higher expression of p‐CREB1 than the matched primary tumor tissues (*P* < 0.05) (Figure 6D). These results demonstrate the clinical importance of p‐CREB1 in ESCC and support the rationale of taking CREB1 as a target for anticancer drug.

### 2.10 CREB1 is Essential for the Suppressive Effect of Imperatorin on TGF*β*2‐ERK Signaling and ESCC Metastasis

To investigate the important role of CREB1 in anticancer effect of imperatorin, CREB1‐knockout cell lines were established based on KYSE150 and KYSE30 by using Clustered Regularly Interspaced Short Palindromic Repeats (CRISPR)/CRISPR‐associated protein 9 (Cas9) technology, designated as KYSE150‐CREB1‐KO and KYSE30‐CREB1‐KO, and a series of functional assays were performed. Unlike CREB1‐expressing cell line (Figure 2C), there was no change of TGF*β*2 and p‐ERK expression in the KYSE150‐CREB1‐KO and KYSE30‐CREB1‐KO cells exposed to imperatorin (Figure S5B, Supporting Information). We found that knockout of CREB1 markedly diminished the suppressive effect of imperatorin on invasion of ESCC cells, confirming that CREB1 is a direct target of imperatorin and mediates its anticancer effect. The CREB1‐deficient KYSE150 and KYSE30 cells were further overexpressed with wild‐type CREB1 and mutant CREB1 (K304E/K305E), respectively, and the results from chamber invasion assay showed that the repressed sensitivity to imperatorin in CREB1‐knockout ESCC cells was markedly restored when the cells were re‐overexpressed with wild‐type CREB1, but not the mutant, to a level comparable to parental cells (Figure 6E). In addition, Western blot data indicated that re‐overexpression of wild‐type CREB1 markedly rescued the inhibitory effect of imperatorin on expression levels of TGF*β*2 and p‐ERK (Figure 6F). Furthermore, the important role of CREB1 in anti‐metastatic activity of imperatorin was evaluated in animal model. KYSE150‐Luc‐CREB1‐KO cells and those further re‐overexpressed with wild‐type and mutant CREB1, respectively, designated as KYSE150‐Luc‐CREB1‐KO/CREB1 WT and KYSE150‐Luc‐CREB1‐KO/CREB1 Mut, as well as parental cells KYSE150‐Luc as control, were intravenously injected into nude mice to establish tumor metastasis. Each group was further divided into two sub‐groups and orally administrated with imperatorin (50 mg kg^−1^) twice a week (Figure 6G). Bioluminescent imaging and histological analysis of the lungs demonstrated that treatment with imperatorin led to a significant decrease of metastatic colonization in the lungs of mice injected with KYSE150‐Luc cells, which is consistent with Figure 2. However, imperatorin could not suppress the ability of CREB1‐knockout cells to form tumor metastasis, and more importantly, the anti‐metastatic effect of imperatorin was recovered when the cells were re‐overexpressed with wild‐type CREB1, but not the mutant CREB1 (Figure 6H; Figure S6, Supporting Information). Overall, as a direct target, CREB1 mediates the anti‐metastatic effect of imperatorin.

## 3 Discussion

Firstly, our findings provided direct evidence suggesting the functional and clinical significance of TGF*β*2 in ESCC metastasis. Although TGF*β* signaling is involved in almost all major cell behaviors, some controversies about the paradoxical roles of TGF*β* in cancer never end.^[^ ^21^, ^22^ ^]^ Moreover, in regards to the role of TGF*β* signaling, most studies focused on the TGF*β*1, but the functional role and clinical relevance of TGF*β*2 are largely unknown. In this study, the data from gain‐ and loss‐of‐function experiments showed that TGF*β*2 positively regulates esophageal cancer invasion (Figure 1; Figure S1, Supporting Information). Expression level of TGF*β*2 was found to be associated with lymph node metastasis and overall survival of the patients with ESCC. To our knowledge, this is the first time to delineate the role of TGF*β*2 in esophageal cancer invasion and metastasis, and support the rationale of TGF*β*2 as a biomarker for cancer diagnosis and prognosis.

Secondly, we identified imperatorin as a novel inhibitor of TGF*β*2 and tumor metastasis. The important role of TGF*β* signaling makes it an attractive target for drug development, and there is an urgent need to screen the therapeutic strategies that can target the TGF*β* signaling pathway without side effects. Some inhibitors of TGF*β* signaling have been developed and tested in preclinical studies,^[^ ^23^ ^]^ but none of them has been approved by U.S. Food and Drug Administration (FDA) due to limited treatment efficacy or obvious toxicity. Here, by screening a bioactive compound library consisting of 429 small molecules, we identified for the first time that imperatorin, a naturally occurring furanocoumarin, exerted a most significantly inhibitory effect on TGF*β*2 expression, and more importantly, markedly suppresses tumor invasion and metastasis in vitro and in vivo without overt side effects (Figure 2). The anticancer effects of imperatorin have been reported in several studies. For example, imperatorin has been reported as a potential chemotherapeutic agent in human larynx cancer.^[^ ^24^ ^]^ Another study showed that imperatorin could suppress drug‐resistant liver cancer through inducing Mcl‐1 degradation to cooperatively trigger Bax translocation and Bak activation.^[^ ^12^ ^]^ However, most of the studies focus on the function of imperatorin in chemoresistance, we provide the first evidence suggesting the potential of imperatorin in suppressing tumor invasion and metastasis.

Quantitative proteomics, IPA analysis and experimental data showed that TGF*β*2 mediated the suppressive effects of imperatorin on ERK signaling and cancer invasion (Figure 3). The efforts to directly target RAS have not been successful so far, although BRAF inhibitors have shown clinical efficacy in patients with RAF‐ and/or RAS‐mutated tumors, acquired resistance remains a great challenge in clinic. Recently, some ERK inhibitors were reported to be effective in overcoming the resistance.^[^ ^25^, ^26^ ^]^ Here, we demonstrated that imperatorin could lead to the inactivation of ERK signaling. Whether imperatorin could be used as adjuvant therapy, in particular, for RAF‐ and/or RAS‐mutated tumors, warrants further investigation.

Thirdly, this study uncovered the direct targeting of CREB1 by imperatorin and the subsequent inhibition of TGF*β*2 transcription. By using SPR and molecular docking analyses, we demonstrated for the first time that imperatorin can directly bind to CREB1 protein, and Lys‐304 and Lys‐305 are important for this interaction. The binding of imperatorin to CREB1 disrupted the interaction of CREB1 with TGF*β*2 promoter, thus repressing the transcription of TGF*β*2 (Figure 5). More importantly, knockout of CREB1 markedly diminished the inhibitory effect of imperatorin on invasion and metastasis of ESCC cells in vitro and in vivo, whereas the repressed sensitivity of CREB1‐knockout ESCC cells to imperatorin was restored upon re‐overexpression of wild‐type CREB1, but not the CREB1 mutant (K304E/K305E) (Figure 6). These data consolidate that CREB1 is essential for anticancer effects of imperatorin. Although CREB1 has been reported to play an important role in tumor development and progression in other cancer types,^[^ ^27^, ^28^, ^29^, ^30^ ^]^ the clinical relevance of CREB1 in ESCC remains unknown. Here, we found that high CREB1 in ESCC is correlated with poor survival of patients, suggesting the rationale of taking CREB1 as a therapeutic target for anti‐metastasis drug. Actually, small molecule inhibitors of CREB1 have been developed,^[^ ^30^, ^31^, ^32^, ^33^, ^34^ ^]^ our findings highlight the potential of imperatorin, which directly targets CREB1, as a therapeutic option for the patients with metastatic ESCC.

Furthermore, this study illustrated the bioactivity of imperatorin in affecting tumor microenvironment. Emerging evidences suggest that cancer metastasis is a complicated process, up to now, the multiple interactions between malignant cells and other cells in tumor microenvironment are far less clear.^[^ ^35^, ^36^ ^]^ Increasing studies documented that fibroblasts, the most abundant component cell type in tumor microenvironment, are capable of interplaying with cancer cells at multiple stages of cancer progression in paracrine manner.^[^ ^37^, ^38^ ^]^ Our previous study suggested that cancer cell‐secreted IGF2 instigates fibroblasts and bone marrow‐derived vascular progenitor cells to promote cancer progression.^[^ ^39^ ^]^ In present study, imperatorin was not only found to attenuate the effects of cancer cells on phenotypes of CAFs through inhibition of TGF*β*2 expression, but also abolish the paracrine effects of fibroblasts on tumor microenvironment, including in vitro and in vivo tumor angiogenesis and cancer invasion (Figure 4). Overall, we revealed that imperatorin not only exerts “cell‐intrinsic” effects to inhibit tumor invasion and metastasis by directly targeting CREB1, but it also exerts “cell‐extrinsic” effects to suppress tumor angiogenesis by inhibiting CAFs‐secreted CCL2.

Taken together, our study uncovers the function of TGF*β*2 in ESCC metastasis, and establishes that imperatorin directly targets and represses transcriptional activity of CREB1 to inhibit TGF*β*2 expression and the subsequent fibroblasts‐secreted CCL2, subsequently, not only inactivates ERK signaling to block cancer cell invasion, but also abrogates the paracrine effects of fibroblasts on tumor angiogenesis and metastasis (Figure 5K). These findings suggest the potential of TGF*β*2 as a biomarker and therapeutic target in ESCC, and provides strong evidence for developing imperatorin as a novel anticancer agent against ESCC metastasis.

## 4 Experimental Section

##### Cell Lines and Drugs

Human ESCC cell lines EC9706,^[^ ^40^ ^]^ and KYSE30, KYSE150 obtained from DSMZ (Braunschweig, Germany),^[^ ^41^ ^]^ were maintained in Dulbecco's Modified Eagle Medium (DMEM) (Life Technologies, Gaithersburg, MD) supplemented with 10% fetal bovine serum (FBS, Life Technologies), 100 µg mL^−1^ streptomycin and 100 µg mL^−1^ penicillin in a humidified atmosphere of 5% CO_2_ at 37  °C. The luciferase‐expressing cell lines KYSE150‐Luc and EC9706‐Luc were generated as previously described.^[^ ^42^ ^]^ Human umbilical vein endothelial cells (HUVECs) obtained from Invitrogen were cultured in M200 medium supplemented with low serum growth supplement (LSGS, Life Technologies) as previously described.^[^ ^43^ ^]^ A drug library consisting of 429 bioactive compounds were obtained from TargetMol (Boston, MA). Recombinant human TGF*β*2 and C‐C motif chemokine ligand 2 (CCL2) purchased from PeproTech (Rocky Hill, NJ), and U0126, imperatorin (Selleck Chemicals, Huston, TX) were diluted in culture medium to obtain the desired concentration.

##### ESCC Tissue and Serum Samples

The human tissue microarrays containing of 186 cases ESCC and 160 cases corresponding adjacent normal tissue (Shanghai Outdo Biotech, Shanghai, China) were used to study TGF*β*2 and p‐CREB1 expression and the correlation with clinicopathological parameters. Another tissue microarray containing 40 pairs of primary ESCC and matched metastatic tumor tissues (Biomax, Rockvile, MD) was included to compare TGF*β*2 and p‐CREB1 expression in primary tumors with that in metastasized tumors of ESCC. Serum samples from 100 ESCC patients and 100 healthy individuals, as well as 12 pairs of ESCC tissues, were collected from affiliated hospitals of Shantou University and Jinan University. Use of all human samples was approved by the committee for ethical review of research involving human subjects at Shantou University and Jinan University, and informed consent was obtained from the patients.

##### Tumor Metastasis Model

The experiment was performed as previously described.^[^ ^44^ ^]^ Briefly, 1 × 10^6^ luciferase‐expressing KYSE150‐Luc and EC9706‐Luc cells were injected intravenously via the tail vein into nude mice and NOD‐Prkdc^em26Cd52^Il2rg^em26Cd22^ (NCG) mice (Nanjing Biomedical Research Institute of Nanjing University, NBRI, Nanjing, China), respectively. One week after cell injection, the mice in treatment group were orally administrated with indicated doses of imperatorin (25 or 50 mg kg^−1^) thrice weekly, and the control group received vehicle only. Metastasis was monitored weekly by bioluminescent imaging (IVIS Lumina Series III Pre‐clinical in vivo imaging system, PerkinElmer, Hopkinton, MA). At the end of the experiment, the lungs, kidney, livers and spleen were dissected and examined histologically for presence of metastasis. Alanine aminotransferase (ALT) and aspartate aminotransferase (AST) in mouse serum were determined using commercial kits (HuiLi Biotech Ltd., Changchun, China). All the animal experiments were approved by the Committee on the Use of Live Animals in Jinan University (No. 20160610204145).

##### Statistical Analysis

All in vitro experiments were repeated at least 3 times. The data were expressed as mean ± SD and compared by *t*‐test. Survival analysis was performed by Kaplan–Meier method with the log‐rank test using the Statistical Package for the Social Sciences (SPSS) (SPSS Inc, Chicago, IL). *P* values < 0.05 were considered as significant for all experiments.

Other detailed information on materials can be found in the Supporting Information and Tables S5–6 in the Supporting Information.

## Conflict of Interest

The authors declare no conflict of interest.

## Author Contributions

W.W.X. and Z.‐H.H. contributed equally to this work. W.W.X., B.L., and Q.‐Y.H. were involved in studying concept and design, drafting of the manuscript, obtaining funding, and studying supervision. W.W.X., Z.‐H.H., L.L., Q.‐H.Z., J.‐Q.L., C.‐C.Z., Y.H., T.‐T.L., Y.W., H.‐F.H., Q.Z. were involved in acquisition of data, analysis and interpretation of data, and statistical analysis. W.‐Y.C., Q.‐S.Y., J.‐F.Z., Y.‐R.Q., L.‐Y.X., E.‐M.L., H.‐X.L were involved in critical revision of the manuscript for important intellectual content, technical and/or material support. All authors commented on the manuscript and approved the final manuscript.

## Supporting information

Supporting Information
