# Supplementary material for: Direct Targeting of CREB1 with Imperatorin Inhibits TGFβ2‐ERK Signaling to Suppress Esophageal Cancer Metastasis
Source: Adv Sci (Weinh). 2020 Jul 1;7(16):2000925. doi: 10.1002/advs.202000925 (PMC7435243; doi:10.1002/advs.202000925)
Supplement: Supplementary file 1 — Supporting Information [file ADVS-7-2000925-s001.pdf]

Supplementary Material and Methods

Isolation of cancer-associated fibroblasts (CAFs)

Cancer-associated fibroblasts (CAF1 and CAF2) were isolated from fresh specimens of primary ESCC tumors as previously described [1]. The fibroblasts used in this study had undergone no more than 10 passages and were maintained in DMEM supplemented with 10% FBS.

Plasmids, transfection, infection and Clustered Regularly Interspaced Short Palindromic Repeats (CRISPR)/CRISPR-associated protein 9 (Cas9) approach-mediated gene knockout

The siRNAs against human TGFβ2, the plasmid expressing human CREB1, and the plasmid expression the sgRNA aginst CREB1 were obtained from TranSheepBio (Shanghai, China). Full-length CREB1 was further cloned into the prokaryotic expression plasmid pGEX-6P-1 (GE Healthcare Life Sciences, Marlborough, MA). The promoter of TGFβ2 was cloned into the pGL3 backbone (Promega, Fitchburg, WI) to generate the pGL3-TGFβ2-pro. Transfection, infection and establishment of stable cell lines were performed as previously described [2]. The CREB1-knockout cell lines were established using sgRNA and CRISPR/Cas9 system, and successful gene knockout was confirmed by DNA sequencing and Western blot. The sequences of siRNA against TGFβ2 and sgRNA against CREB1 were listed in Supplementary Table 5. The sequences for subcloning of CREB1 to pGEX-6P-1 and cloning of TGFβ2 promoter were listed in Supplementary Table 6.

Cell viability assay

A WST-1 Cell Proliferation and Cytotoxicity Assay Kit (Beyotime Biotechnology, Shanghai, China) was used to determine cell viability. Cells were seeded in the 96-well plate and treated with increasing concentrations of imperatorin for indicated time, followed by incubation with WST-1 at 37 °C for 2 h. The absorbance was measured on an automated microplate spectrophotometer (BioTek Instruments, Winooski, VT) at 450 nm.

Transwell cell migration/invasion assay

The motility of cells was monitored with the use of migration/invasion chamber as previously described [3]. The invasion chamber is coated with matrigel (BD Biosciences, Bedford, MA).The cells suspended in serum-free medium were loaded into the upper compartment of the chamber, and complete or conditioned media were used as chemoattractant in the lower chamber. The migrated/invaded cells were fixed with methanol and stained with crystal violet (0.1%).

Tube formation assay

The angiogenic activity of endothelial cells was measured *in vitro* using tube formation assay as previously described [4]. In brief, HUVECs were seeded in a 96-well plate pre-coated with matrigel (BD Biosciences). After 7 h, capillary-like tubes were imaged and the length of tube formation was quantified in six random

fields from each well using the software Stereo Investigator (MBF Bioscience, VT).

Stable Isotope Labeling by Amino Acids in Cell Culture (SILAC) labeling, protein digestion and mass spectrometry analyses

KYSE150 cells were cultured in SILAC RPMI 1640 media (Thermo Fisher Scientific, San Jose, CA) for seven cell doublings, supplemented with 10% dialytic fetal bovine serum (Life Technologies) and either “light media” (Arg0, Lys0) or “heavy media” (Arg10, Lys8; Cambridge Isotope Laboratories, Andover, MA), respectively. The “light” labeled KYSE150 cells were treated with 40 μM imperatorin for 24 h, and the “heavy” labeled KYSE150 cells were treated with DMSO. A total of 500 μg “heavy” and 500 μg “light” protein were mixed together, and protein digestion and mass spectrometry analysis were performed as described previously [5]. Peptides were analyzed by triple-TOF 5600 mass spectrometer (AB SCIEX, Framingham, CA) according to the manufacturer’s instructions. Protein identification and quantification were performed by MaxQuant software (v. 1.5.2.8) against the UniProt-Swiss Human database (2017_08 release, 20 237 entries). Protein and peptide FDRs were set to 1%, and the normalized ratio of “heavy” versus “light” was calculated by MaxQuant.

Nuclear and cytosolic protein extraction

The cytosolic and nuclear fractions were isolated as previously described [6]. In brief, cells were collected and resuspended in 500 μL extraction buffer (10 mM HEPES-KCl [pH 7.6], 10 mM KCl, 5 mM $MgCl_2$), and then incubated on ice for 10

min. Next, 500 μL extraction buffer with 1% Triton-100 was added to the cell supernatant to solubilize plasma membrane and leave the nuclear membrane intact. To obtain the nuclear pellet, the cell supernatant was incubated on ice for 20 min, and 500 μL nuclear isolation buffer (10 mM HEPES-KCl [pH 7.6], 10 mM KCl, 5 mM $MgCl_2$, sucrose) was added, and then the homogenates were centrifuged at 600 g for 10 min at 4 °C. The supernatant fraction is the cytosolic fraction, and the pellet fraction is the enriched nuclear fraction.

Western blot

Cells were lysed in lysis buffer (Cell Signaling Technology, Beverly, MA) according to the manufacturer's instructions, and a BCA kit (Thermo Fisher Scientific) was used to determine the protein concentration [7]. The proteins were separated by SDS-polyacrylamide gel electrophoresis and transferred to a PVDF membrane (Millipore, Bedford, MA). After blocking with 5% nonfat milk for 1 h, the membrane was incubated with primary antibody at 4°C overnight, and washed with Tris-Buffered Saline Tween-20 (TBST) followed by a incubation with the corresponding HRP-conjugated secondary antibodies (Cell Signaling Technology) at room temperature for 1 h. The reaction was visualized using ECL (Bio-Rad, Hercules, CA) and detected by exposure to autoradiographic film. The primary antibodies used included TGFβ2 and CCL2 from Proteintech (Rosemont, IL), E-cadherin and fibronectin from BD Biosciences, N-cadherin, snail, p-ERK, ERK, p-CREB1, CREB1, Eight twenty one protein (ETO), p-smad3 and smad3 obtained from Cell Signaling

Technology company, fibroblasts activation protein (FAP; Abcam, Cambridge, UK), and alpha smooth muscle actin (α-SMA; Sigma, St Louis, MO), specificity protein 1 (SP1), Regulatory Factor X1 (RFX1) and actin purchased from Santa Cruz Biotechnology (Santa Cruz, CA).

Enzyme-Linked Immunosorbent Assay (ELISA)

Human TGFβ2 and CCL2 ELISA Kit (RayBiotech, Norcross, GA) was used to determine the concentration of TGFβ2 and CCL2 in the conditioned medium or human serum samples, respectively, according to the manufacturer' instructions.

Quantitative real-time polymerase chain reaction (qRT-PCR)

Total RNA was isolated using Trizol reagent according to the manufacturer's protocol (Life Technologies). RNA was converted to cDNA using PrimeScript II first Strand cDNA Synthesis Kit (Takara, Dalian, China) and the subsequent quantitative PCR was performed on a Bio-Rad Mini Opticon real-time PCR system using SYBR Premix Ex *TaqII* (Takara) according to the manufacturer's instructions. Actin was included as internal control. The primers were listed as Supplementary Table 2.

Molecular Docking and molecular dynamics analysis

The nuclear magnetic resonance (NMR) structure of CREB1 was downloaded from RCSB Protein Data Bank (PDB code: 2LXT), and the imperatorin structure was

constructed using UCSF Chimera [8]. DOCK 6.7 program was then utilized to conduct semi-flexible docking where 10000 different orientations were generated [9, 10]. The clustering analysis was performed to obtain the best scored poses, with a RMSD threshold of 2.0 Å. All molecular dynamics simulations were performed using GROMACS version 2016.4 [11] with AMBER ff99SB-ILDN force filed. CREB1-imperatorin complexes were centered in a cubic box of 10 Å solvated using TIP3P water model and SPC216 solvent configuration. MM/PBSA method was employed to calculate the binding free energies of ligand and protein as decribed previously [12].

Site-directed mutagenesis and luciferase reporter assay

The mutant constructs for pGL3-TGFβ2-pro and pGEX-6P-1-CREB1 were generated using the Fast Mutagenesis System Kit (TransGen Biotech, Beijing, China) according to the manufacturer's protocol [13]. The primers used for generating pGL3-TGFβ2-pro-Mut#1, pGL3-TGFβ2-pro-Mut#2, pGEX-6P-1-CREB1(K304E) and pGEX-6P-1-CREB1(K305E) were listed in the Supplementary Table 2. Luciferase activity was measured by using Dual-luciferase Reporter Assay System (Promega) as previously described [14].

Chromatin immunoprecipitation (ChIP)-quantitative PCR

The ChIP assay was performed as previously described by using simple CHIP enzymatic chromatin IP kit (Cell Signaling) according to the manufacturer’s manual

[15]. In brief, *in vivo* protein and DNA crosslinking was performed using 37% formaldehyde, followed by sonication and chromatin digestion. The protein-DNA complexes were immunoprecipitated using CREB1 antibody or negative control IgG antibody, and the purified DNA was subjected to SYBR Green PCR analysis (Takara). Relative expression was calculated using the comparative Ct method after normalization to GAPDH control.

Protein purification and surface plasmon resonance (SPR) assay

Protein was purified by using Glutathione S-transferase (GST) tag protein purification kit (Beyotime Biotechnology). The pGEX-6P-1 plasmid expressing GST-tagged wild-type or mutant CREB1 was transformed into E. coli BL21 star (DE3) cells, and isopropyl β-D-thiogalactopyranoside (IPTG) was added when bacteria culture were grown to a 600 nm (OD600) optical density of about 0.6. After obtaining CREB1-GST protein, the GST tag was cut with PreScission Protease (Beyotime Biotechnology) according to manufacture's protocol. SPR analysis was performed using the Biacore X100 system (GE Healthcare Life Sciences). Wide type CREB1 and mutant CREB1 protein was immobilized by amine coupling onto a CM7 chip (GE Healthcare Life Sciences) as described previously (8). Imperation in PBS buffer was added with a speed of 30 μL/min for 90 s, and dissociation was evaluated by passing HBS buffer alone over the chip at 30 μl/min for 10 min.

Tumor xenograft model

Female BALB/c nude mice aged 6-8 weeks were maintained under standard conditions and cared for according to the institutional guidelines for animal care. All the animal experiments were approved by the Committee on the Use of Live Animals in Jinan University. ESCC cells were subcutaneously injected into flanks of mice, and the mice were randomized into treatment and control groups when the tumors reached ~5 mm diameter. The treatment group received oral gavage of imperatorin (50 mg/kg dissolved in corn oil) thrice weekly, whereas the control group received the vehicle only. A sub-group of imperatorin-treated mice also received twice weekly intravenous injections of CCL2 recombinant protein (300 ng/mouse). At the end of the experiment, the tumors were collected for immunohistochemical analysis of CD31 as described previously [16].

Immunohistochemistry

Paraffin-embedded sections were deparaffinized and rehydrated, followed by antigen retrieval for 15 min in 10 mM citrate buffer (pH 6). After blocking with normal serum, the slides were incubated with primary antiobdy at 4°C overnight, and then incubated with corresponding biotinylated secondary antibody. After incubation with peroxidase-conjugated avidin-biotincomplex, immunostaining was visualized using 3, 3’-diaminobenzidine (Dako, Mississauga, ON, Canada) as chromogen, and then the slides were counterstained with hematoxylin. The degree of immunostaining of sections was reviewed and independently scored by two pathologists. The scores based on the intensity of staining were graded into 1-4 as follows: representing

negative (1), weak (2), moderate (3) and strong (4) staining. Specimens assigned scores of 1 to 2 were considered low expression, whereas those with scores 3 to 4 were regarded as having high expression. Microvessel density was calculated as the mean number of CD31-positive vessels in six random fields from representative tumor sections [17].

Analysis of gene expression in The Cancer Genome Atlas (TCGA) database

Gene expression datasets from patients with esophageal cancer were downloaded from TCGA， and gene profiles in tumors of patients without lympy node metastasis (N0) were compared with those with high lymph node metastasis (N3). The differentially expressed genes (fold change > 1.2) were subjected to ClueGO (v2.5.2), a widely used plugin of Cytoscape (v3.6.0) [18, 19], for Gene Ontology (GO) analysis. The *P* values of gene enrichment in GO terms were calculated based on hypergeometric distribution, and the result of bar plot only showed the GO terms with *P* values < 0.00001, while the pie plot was calculated based on the total GO terms.

References

1. Xu WW, Li B, Guan XY, Chung SK, Wang Y, Yip YL, Law SY, Chan KT, Lee NP, Chan KW, et al: **Cancer cell-secreted IGF2 instigates fibroblasts and bone marrow-derived vascular progenitor cells to promote cancer progression.** *Nat Commun* 2017, **8:**14399.

2. Xu WW, Li B, Zhao JF, Yang JG, Li JQ, Tsao SW, He QY, Cheung ALM: **IGF2 induces CD133 expression in esophageal cancer cells to promote cancer stemness.** *Cancer Lett* 2018, **425:**88-100.

3. Xu WW, Zheng CC, Huang YN, Chen WY, Yang QS, Ren JY, Wang YM, He QY, Liao HX, Li B: **Synephrine Hydrochloride Suppresses Esophageal Cancer Tumor Growth and Metastatic Potential through Inhibition of Galectin-3-AKT/ERK Signaling.** *J Agric Food Chem* 2018, **66:**9248-9258.

4. Xu WW, Li B, Lam AK, Tsao SW, Law SY, Chan KW, Yuan QJ, Cheung AL: **Targeting VEGFR1- and VEGFR2-expressing non-tumor cells is essential for esophageal cancer therapy.** *Oncotarget* 2015, **6:**1790-1805.

5. Wang Y, Zhang J, Huang ZH, Huang XH, Zheng WB, Yin XF, Li YL, Li B, He QY: **Isodeoxyelephantopin induces protective autophagy in lung cancer cells via Nrf2-p62-keap1 feedback loop.** *Cell Death Dis* 2017, **8:**e2876.

6. Wang Y, Yu RY, Zhang J, Zhang WX, Huang ZH, Hu HF, Li YL, Li B, He QY: **Inhibition of Nrf2 enhances the anticancer effect of 6-O-angeloylenolin in lung adenocarcinoma.** *Biochem Pharmacol* 2017, **129:**43-53.

7. Hu HF, Xu WW, Wang Y, Zheng CC, Zhang WX, Li B, He QY: **Comparative**

Proteomics Analysis Identifies Cdc42-Cdc42BPA Signaling as Prognostic Biomarker and Therapeutic Target for Colon Cancer Invasion. *J Proteome Res* 2018, **17:**265-275.

8. Pettersen EF, Goddard TD, Huang CC, Couch GS, Greenblatt DM, Meng EC, Ferrin TE: **UCSF Chimera--a visualization system for exploratory research and analysis.** *J Comput Chem* 2004, **25:**1605-1612.

9. Lang PT, Brozell SR, Mukherjee S, Pettersen EF, Meng EC, Thomas V, Rizzo RC, Case DA, James TL, Kuntz ID: **DOCK 6: combining techniques to model RNA-small molecule complexes.** *RNA* 2009, **15:**1219-1230.

10. Mukherjee S, Balius TE, Rizzo RC: **Docking validation resources: protein family and ligand flexibility experiments.** *J Chem Inf Model* 2010, **50:**1986-2000.

11. Pronk S, Pall S, Schulz R, Larsson P, Bjelkmar P, Apostolov R, Shirts MR, Smith JC, Kasson PM, van der Spoel D, et al: **GROMACS 4.5: a high-throughput and highly parallel open source molecular simulation toolkit.** *Bioinformatics* 2013, **29:**845-854.

12. Kumari R, Kumar R, Open Source Drug Discovery C, Lynn A: **g_mmpbsa--a GROMACS tool for high-throughput MM-PBSA calculations.** *J Chem Inf Model* 2014, **54:**1951-1962.

13. Zhong Y, Yang J, Xu WW, Wang Y, Zheng CC, Li B, He QY: **KCTD12 promotes tumorigenesis by facilitating CDC25B/CDK1/Aurora A-dependent G2/M transition.** *Oncogene* 2017, **36:**6177-6189.

14. Li B, Xu WW, Guan XY, Qin YR, Law S, Lee NP, Chan KT, Tam PY, Li YY, Chan KW, et al: **Competitive Binding Between Id1 and E2F1 to Cdc20 Regulates E2F1 Degradation and Thymidylate Synthase Expression to Promote Esophageal Cancer Chemoresistance.** *Clin Cancer Res* 2016, **22:**1243-1255.

15. Li B, Hong P, Zheng CC, Dai W, Chen WY, Yang QS, Han L, Tsao SW, Chan KT, Lee NPY, et al: **Identification of miR-29c and its Target FBXO31 as a Key Regulatory Mechanism in Esophageal Cancer Chemoresistance: Functional Validation and Clinical Significance.** *Theranostics* 2019, **9:**1599-1613.

16. Li B, Tsao SW, Li YY, Wang X, Ling MT, Wong YC, He QY, Cheung AL: **Id-1 promotes tumorigenicity and metastasis of human esophageal cancer cells through activation of PI3K/AKT signaling pathway.** *Int J Cancer* 2009, **125:**2576-2585.

17. Li B, Li YY, Tsao SW, Cheung AL: **Targeting NF-kappaB signaling pathway suppresses tumor growth, angiogenesis, and metastasis of human esophageal cancer.** *Mol Cancer Ther* 2009, **8:**2635-2644.

18. Bindea G, Mlecnik B, Hackl H, Charoentong P, Tosolini M, Kirilovsky A, Fridman WH, Pages F, Trajanoski Z, Galon J: **ClueGO: a Cytoscape plug-in to decipher functionally grouped gene ontology and pathway annotation networks.** *Bioinformatics* 2009, **25:**1091-1093.

19. Shannon P, Markiel A, Ozier O, Baliga NS, Wang JT, Ramage D, Amin N,

Schwikowski B, Ideker T: **Cytoscape: a software environment for integrated models of biomolecular interaction networks.** *Genome Res* 2003, **13:**2498-2504.

Supplementary Figure Legends

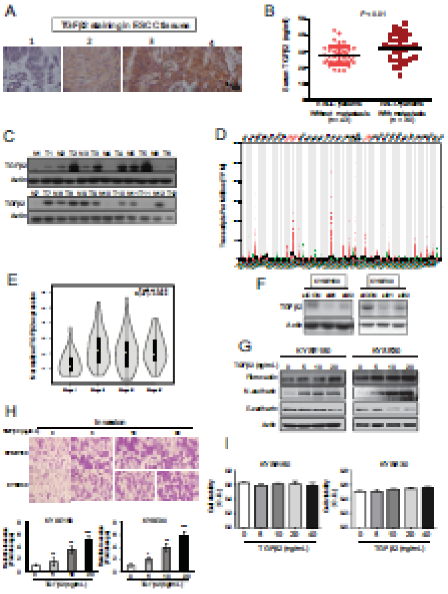

Supplementary Figure 1

Supplementary Figure 1. (A) Representative images of ESCC with immunohistochemical staining scores of 1-4 for TGFβ2. **(B)** TGFβ2 level in the serum of 43 cases ESCC patients without metastasis and 30 cases ESCC patients with metastasis. **(C)** Western blot analysis of TGFβ2 expression in 12 pairs of ESCC tumor and matched normal tissue. **(D)** The GEPIA database was used to analyze the gene expression of TGFβ2 in multiple cancer types. **(E)** The relationship between TGFβ2 expression and pathological stages of esophageal cancer via the GEPIA database. Note that TGFβ2 expression is increased in advanced stages of tumors. **(F)** Successful

knockdown of TGFβ2 in KYSE150 and KYSE30 cells by using siRNA. **(G-I)** KYSE150 and KYSE30 cells were exposed to increasing doses of TGFβ2 (0, 5, 10, 20 ng/ml) for 24 h, and then expression levels of Fibronectin, N-cadherin and E-cadherin were detected by Western blot **(G)**, invasive ability was examined by Boyden chamber assay **(H)**, and cell viability was determined by WST-1 assay **(I)**. Bars, SD; *, *P* < 0.05; **, *P* < 0.01; ***, *P* < 0.001.

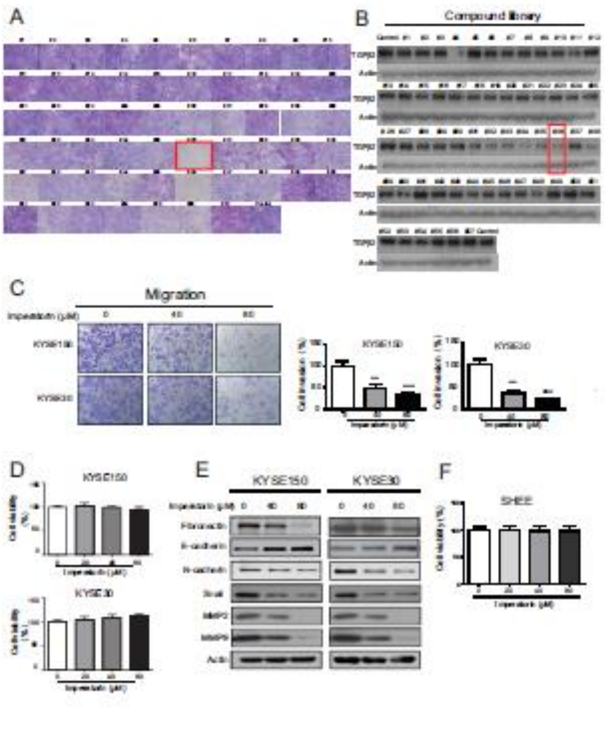

Supplementary Figure 2

Supplementary Figure 2. (A) Boyden chamber invasion assay showing the effect of 57 candidate compounds on invasion of KYSE150 cells. **(B)** Western blot comparing the effect of 57 candidate compounds on the expression of TGFβ2 in KYSE150 cells. **(C)** Chamber migration assay showing the effect of imperatorin on cell migration. **(D-E)** KYSE150 and KYSE30 cells were treated with increasing concentrations of

imperatorin (0, 20, 40, 80 μM) for 24 h, and cell viability was determined by WST-1 assay **(D)**, and Western blot was performed to compare expression of Fibronectin, E-cadherin, N-cadherin, Snail, MMP2 and MMP9 **(E)**. **(F)** WST-1 assay showing that imperatorin had no toxicity on normal esophageal epithelial cells. Bars, SD.

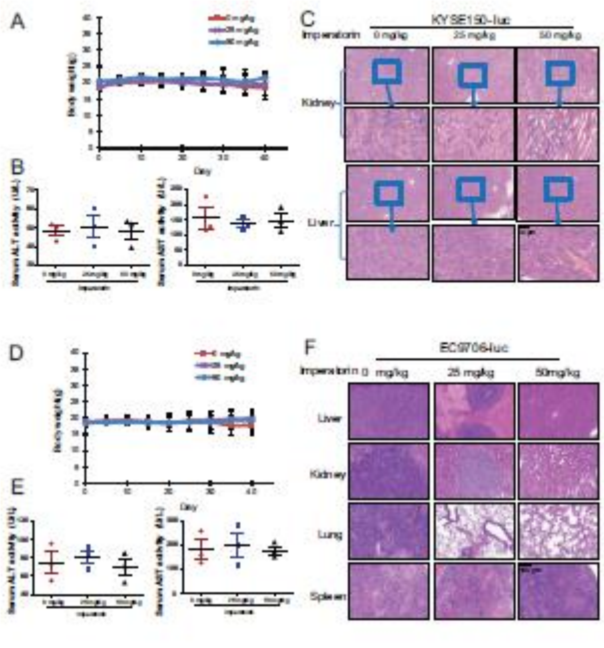

Supplementary Figure 3

Supplementary Figure 3. (A) The body weight of nude mice in treatment and control groups was monitored. **(B)** The alanine aminotransferase (ALT) and aspartate aminotransferase) (AST) level was determined in the nude mice of treatment and control groups. **(C)** H & E staining of liver and kidney in the nude mice treated with imperatorin (25mg/kg or 50mg/kg) and vehicle, respectively. **(D)** The body weight of NCG mice among different groups was compared. **(E)** No significant difference in ALT and AST level was detected in the NCG mice treated with imperatorin or vehicle.

(F) H & E staining showing the metastatic niches in lung, liver, kidney and spleen. Bars, SD.

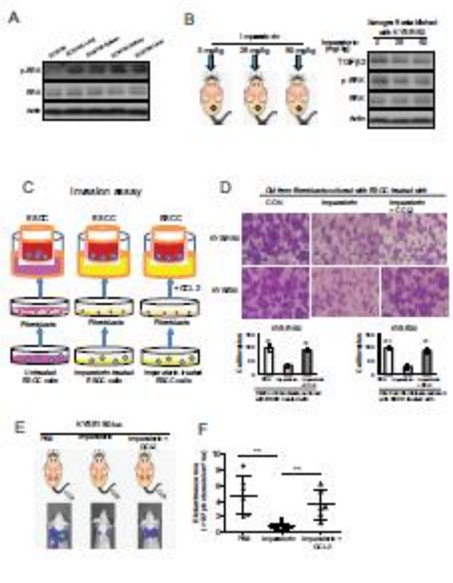

Supplementary Figure 4

Supplementary Figure 4. (A) The expression of p-ERK and ERK was determined in multi-metastatic cell lines and parental cell line by Western blot. **(B)** The expression of TGFβ2，p-ERK and ERK was determined in the tumor xenografts by Western blot. **(C)** Diagram showing the approach of collecting CM from imperatorin-treated or untreated ESCC cells to study the paracrine effect of CAFs on invasion of cancer cells in presence or absence of CCL2. **(D)** Boyden chamber showing the invasion of ESCC cells attracted by the indicated different CM from CAFs with or without addition of CCL2 (5 ng/ml). **(E-F)** Diagram showing the approach to establish the *in vivo* model. Bioluminescence imaging and quantification of metastasis as indicated. Bars, SD; *, $P < 0.05$; **, $P < 0.01$; ***, $P < 0.001$.

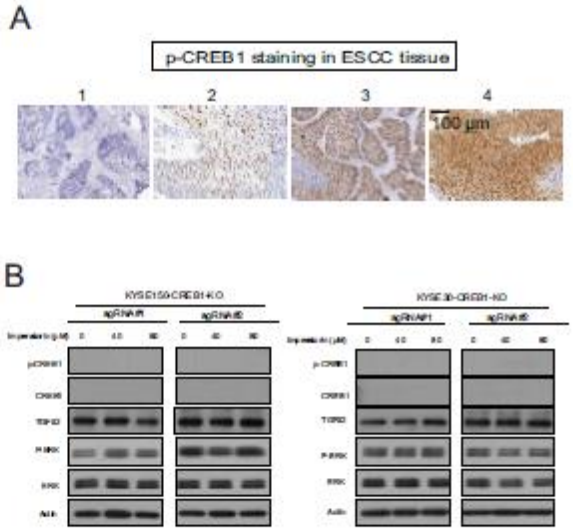

Supplementary figure 5

Supplementary Figure 5. (A) Representative images of ESCC with immunohistochemical staining scores of 1-4 for p-CREB1. **(B)** Western blot showing the effect of imperatorin on TGFβ2 and p-ERK expression in KYSE150-CREB1-KO and KYSE30-CREB1-KO cells.

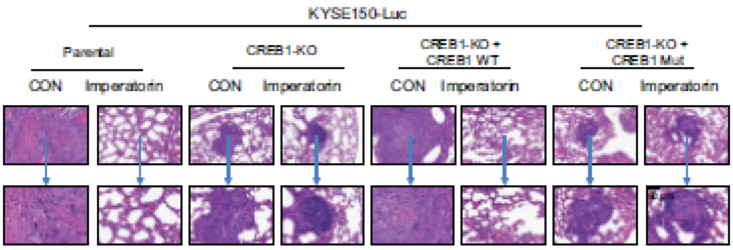

Supplementart Figure 6

Supplementary Figure 6. H & E staining of lungs in the different groups of nude mice intravenously injected with the indicated cells and treated with imperatorin or vehicle.

Supplementary Table 1. Clinicopathological information of 186 cases patients with esophageal cancer.

	**Survival time (Months)**	**Status**	**Gender**	**Age**	**T**	**N**	**M**
1	31	Dead	Male	65	T3	N0	M0
2	10	Dead	Male	56	T3	N1	M0

3	19	Dead	Male	60	T3	N1	M0
4	10	Dead	Male	64	T3	N0	M0
5	9	Dead	Male	59	T3	N0	M0
6	8	Dead	Male	57	T2	N1	M0
7	27	Dead	Female	58	T3	N1	M0
8	58	Dead	Male	70	T3	N0	M0
9	26	Dead	Male	73	T3	N1	M0
10	13	Dead	Female	74	T3	N1	M0
11	97	Live	Male	66	T3	N0	M0
12	15	Dead	Male	73	T3	N0	M0
13	96	Live	Female	74		N0	M0
14	60	Dead	Male	78		N1	M0
15	23	Dead	Female	52	T3	N1	M0
16	4	Dead	Male	73	T2	N0	M0
17	31	Dead	Male	75	T3	N0	M0
18	51	Dead	Male	51	T3	N0	M0
19	47	Dead	Male	51	T3	N2	M0
20	29	Dead	Male	73		N0	M0
21	10	Dead	Male	59	T3	N2	M0
22	19	Dead	Female	73	T3	N0	M0
23	22	Dead	Male	63	T3	N1	M0
24	35	Dead	Male	51	T3	N2	M0
25	10	Dead	Male	60	T3	N2	M0
26	90	Live	Female	51	T3		
27	8	Dead	Female	50	T3	N0	M0
28	23	Dead	Male	71	T3	N2	M0
29	9	Dead	Male	51	T2	N0	M0
30	6	Dead	Male	64	T4	N3	M0
31	43	Dead	Male	64	T3	N0	M0
32	44	Dead	Male	55	T2	N0	M0
33	8	Dead	Male	52	T3	N1	M0
34	9	Dead	Male	59	T3	N3	M0
35	16	Dead	Male	70	T3	N1	M0
36	87	Live	Male	65	T3	N0	M0
37	26	Dead	Male	63	T2	N0	M0
38	87	Live	Female	56	T3	N0	M0
39	10	Dead	Female	67	T3	N0	M0
40	43	Dead	Male	48	T3	N1	M0
41	44	Dead	Male	53	T3	N1	M0
42	85	Live	Female	71	T3	N0	M0
43	23	Dead	Male	69	T3	N1	M0
44	73	Live	Male	61	T3	N1	M0
45	4	Dead	Male	77	T3	N0	M0

46	9	Dead	Male	61	T3	N1	M0
47	13	Dead	Male	59	T3	N0	M0
48	15	Dead	Female	56	T4	N0	M0
49	13	Dead	Female	68	T3	N0	M0
50	2	Dead	Male	77	T3	N0	M0
51	78	Live	Male	57	T2	N1	M0
52	33	Dead	Male	61	T3	N0	M0
53	12	Dead	Male	68	T3	N3	M0
54	12	Dead	Female	72	T3	N0	M0
55	78	Live	Male	57	T3	N0	M0
56	4	Dead	Male	74	T3	N0	M0
57	5	Dead	Male	72	T2	N2	M0
58	30	Dead	Female	65	T3	N0	M0
59	77	Live	Male	75	T3	N0	M0
60	22	Dead	Female	63	T3	N0	M0
61	5	Dead	Female	73	T3	N2	M0
62	0	Dead	Male	76	T3	N0	M0
63	5	Dead	Male	61	T3	N1	M0
64	15	Dead	Male	79	T2	N3	M0
65	5	Dead	Male	72	T3	N1	M0
66	16	Dead	Male	54	T3	N1	M0
67	74	Live	Male	76	T2	N1	M0
68	26	Dead	Male	65	T3	N3	M0
69	74	Live	Female	81	T2	N0	M0
70	74	Live	Female	68	T3	N1	M0
71	1	Dead	Male	81	T3	N1	M0
72	5	Dead	Male	64	T3	N1	M0
73	73	Live	Female	71	T1	N0	M0
74	15	Dead	Male	48	T3	N2	M0
75	55	Dead	Male	76	T1	N0	M0
76	73	Live	Male	52	T2	N1	M0
77	25	Dead	Female	68	T3	N1	M0
78	72	Live	Female	74	T1	N0	M0
79	71	Live	Male	63	T1	N0	M0
80	10	Dead	Male	52	T3	N1	M0
81	15	Dead	Female	67	T3	N2	M0
82	24	Dead	Male	72	T3	N0	M0
83	6	Dead	Male	69	T3	N1	M0
84	2	Dead	Male	72	T3	N2	M0
85	27	Dead	Male	74	T3	N2	M0
86	33	Dead	Male	73	T3	N0	M0
87	6	Dead	Male	51	T3	N0	M0
88	8	Dead	Male	49	T3	N2	M0

89	2	Dead	Female	82	T3	N2	M0
90	15	Dead	Male	65	T3	N1	M0
91	5	Dead	Male	80	T3	N1	M0
92	76	Live	Female	79	T3	N0	M0
93	7	Dead	Male	50	T3	N2	M0
94	14	Dead	Male	54	T4	N2	M0
95	73	Live	Female	74	T3	N0	M0
96	4	Dead	Male	62	T3	N2	M0
97	10	Dead	Female	74	T3	N0	M0
98	10	Dead	Male	74	T3	N2	M0
99	27	Dead	Male	79	T3	N1	M0
100	28	Dead	Male	65	T3	N1	M0
101	20	Dead	Male	69	T3	N3	M0
102	10	Dead	Male	80	T3	N0	M0
103	78	Live	Male	52	T2	N0	M0
104	77	Live	Male	57	T3	N0	M0
105	8	Dead	Male	60	T3	N0	M0
106	17	Dead	Male	77	T3	N0	M0
107	8	Dead	Male	65		N1	M0
108	75	Live	Male	76	T2	N0	M0
109	75	Live	Female	58	T3	N1	M0
110	2	Dead	Male	60	T3	N2	M0
111	8	Dead	Male	80	T3	N0	M0
112	75	Live	Male	57	T3	N1	M0
113	28	Dead	Male	72	T3	N0	M0
114	6	Dead	Male	64	T3	N1	M0
115	27	Dead	Male	60	T3	N0	M0
116	62	Dead	Male	61	T2	N0	M0
117	7	Dead	Male	69	T2	N0	M0
118	73	Live	Female	75	T2	N0	M0
119	20	Dead	Male	61	T3	N0	M0
120	5	Dead	Male	84	T3	N0	M0
121	72	Live	Female	63	T2	N1	M0
122	25	Dead	Male	65	T2	N0	M0
123	38	Dead	Male	56	T3	N1	M0
124	51	Dead	Male	81	T3	N0	M0
125	3	Dead	Male	49	T3	N3	M0
126	72	Live	Female	68	T2	N0	M0
127	72	Live	Male	52	T2	N0	M0
128	14	Dead	Male	66	T3	N1	M0
129	71	Live	Male	65	T3	N2	M0
130	71	Live	Female	81	T1	N0	M0
131	16	Dead	Female	67	T3	N0	M0

132	2	Dead	Male	71	T3	N1	M0
133	15	Dead	Male	55	T3	N2	M0
134	0.13	Dead	Female	63	T1	N0	M0
135	69	Live	Male	73	T2	N0	M0
136	21	Dead	Male	67	T2	N2	M0
137	9	Dead	Male	63	T2	N0	M0
138	68	Live	Male	66	T3	N0	M0
139	33	Dead	Male	51	T3	N2	M0
140	12	Dead	Male	69	T2	N2	M0
141	66	Live	Female	72	T3	N1	M0
142	11	Dead	Male	72	T3	N0	M0
143	42	Dead	Male	63	T3	N0	M0
144	1	Dead	Female	78	T3	N0	M0
145	3	Dead	Male	59	T3	N2	M0
146	8	Dead	Male	57	T3	N2	M0
147	26	Dead	Male	74	T3	N1	M0
148	63	Live	Male	69		N1	M0
149	63	Live	Male	62	T3	N0	M0
150	25	Dead	Female	79	T2	N1	M0
151	11	Dead	Female	65	T3	N1	M0
152	11	Dead	Male	83		N2	M0
153	16	Dead	Male	62		N2	M0
154	62	Live	Male	69		N0	M0
155	62	Live	Male	63		N1	M0
156	30	Dead	Male	72		N0	M0
157	8	Dead	Male	77	T3	N3	M0
158	2	Dead	Male	65	T2	N2	M0
159	4	Dead	Male	75	T3	N1	M0
160	12	Dead	Male	69	T3	N0	M0
161	18	Dead	Male	76	T3	N1	M0
162	16	Dead	Male	73	T3	N3	M0
163	9	Dead	Female	73	T2	N1	M0
164	6	Dead	Female	62	T3	N0	M0
165	3	Dead	Male	60	T3	N0	M0
166	60	Live	Male	51	T3	N1	M0
167	59	Live	Female	69	T3	N1	M0
168	59	Live	Male	50	T3	N0	M0
169	19	Dead	Male	69	T3	N2	M0
170	16	Dead	Male	50	T3	N2	M0
171	58	Live	Female	80	T1	N0	M0
172	15	Dead	Male	64	T3	N1	M0
173	10	Dead	Male	59	T3	N1	M0
174	56	Live	Male	63	T3	N0	M0

175	56	Live	Male	52	T3	N2	M0
176	10	Dead	Male	58	T4	N2	M0
177	55	Live	Female	75	T2	N1	M0
178	1	Dead	Male	61	T3	N1	M0
179	1	Dead	Male	66	T4b	N2	M0
180	55	Live	Male	78	T3	N0	M0
181	11	Dead	Male	57	T3	N2	M0
182	31	Dead	Male	64	T3	N0	M0
183	28	Dead	Male	62	T3	N0	M0
184	19	Dead	Male	85	T3	N0	M0
185	9	Dead	Male	60	T3	N1	M0
186	12	Dead	Male	54	T3	N1	M0

Supplementary Table 2. The inhibitory rate of 57 candidate compounds.

Case#	Name	CAS	Average inhibitory rate (%)	SD
1	Mandelic acid	611-71-2	-1.04	4.95
2	Orcinol	504-15-4	1.47	9.69
3	Sakakin	21082-33-7	1.20	4.31
4	Glucosylvitexin	76135-82-5	1.47	3.63
5	2-(4-Hydroxyphenyl)ethanol	501-94-0	3.11	3.85
6	Isovanillin	621-59-0	9.22	5.24
7	(-)-Bilobalide	33570-04-6	2.95	2.11
8	Nodakenin	495-31-8	1.47	3.85
9	Elemicin	487-11-6	0.82	1.36
10	Panaxatriol	32791-84-7	1.86	4.10
11	Angelic acid	565-63-9	2.40	4.43
12	Rebaudioside A	58543-16-1	2.18	2.98
13	Methyl 4-hydroxycinnamate	3943-97-3	0.66	2.11
14	Nardosinone	23720-80-1	4.59	2.95
15	Coixol	532-91-2	1.31	4.12
16	2-Methoxycinnamic acid	6099-03-2	3.82	2.18
17	Mogroside V	88901-36-4	1.09	1.56
18	Vitexin-2"-O-rhamnoside	64820-99-1	7.75	2.46
19	Ginkgolide C	15291-76-6	3.11	1.56
20	Daurisoline	70553-76-3	0.93	4.56
21	Oxysophocarpine	26904-64-3	2.84	3.04
22	Sophoricoside	152-95-4	2.46	4.62
23	Neosperidin dihydrochalcone	20702-77-6	1.20	2.88
24	Isochlorogenic acid C	32451-88-0	2.02	3.07
25	2-Pentylfuran	3777-69-3	6.39	1.64

26	4-(4-Hydroxyphenyl)-2-butanone	5471-51-2	8.84	4.86
27	Echinatin	34221-41-5	23.25	14.95
28	Pseudoginsenoside F11	69884-00-0	4.80	3.37
29	Helicid	80154-34-3	21.23	14.28
30	Methyl hesperidin	11013-97-1	8.57	1.64
31	trans-4-Hydroxycinnamic acid	7400-08-0	10.26	3.20
32	Methyl syringate	884-35-5	8.52	3.77
33	Shionone	10376-48-4	3.98	1.67
34	Ethyl coumarin-3-carboxylate	1846-76-0	7.70	1.09
35	Phlorizin dihydrate	7061-54-3	9.39	1.16
36	Imperatorin	482-44-0	77.18	7.49
37	7-hydroxy-4-methyl-8-nitrocoumarin	19037-69-5	7.97	3.36
38	4-Methyl-6,7-dihydroxycoumarin	529-84-0	10.26	5.12
39	Ethyl Vanillate	617-05-0	1.26	4.89
40	Perillartine	30950-27-7	65.94	7.74
41	trans-4-phenylbut-3-en-2-one	1896-62-4	50.05	3.44
42	20R-Ginsenoside Rg2	80952-72-3	15.99	2.54
43	Macranthoidin A	140360-29-8	19.71	4.45
44	Jujuboside A	55466-04-1	22.38	8.85
45	Liensinine perchlorate	2385-63-9	18.94	26.81
46	Synephrine HCL	5985-28-4	74.78	7.77
47	Synephrine	94-07-5	41.21	8.68
48	Sinapine thiocyanate	7431-77-8	42.36	6.06
49	Macranthoidin B	136849-88-2	4.04	2.74
50	Rebaudioside C	63550-99-2	4.26	3.88
51	Xanthoxylin	90-24-4	-0.38	1.34
52	Glycyrrhizic acid ammonium salt	53956-04-0	28.93	11.22
53	L-Carnitine inner salt	541-15-1	23.47	7.93
54	Hordenine	539-15-1	16.21	1.98
55	3,4,5-Trimethoxycinnamic acid	90-50-6	24.40	5.07
56	7-Methoxy-4-methylcoumarin	2555-28-4	53.60	10.57
57	Byakangelicin	19573-01-4	5.13	3.84

Supplementary Table 3. The differentiately expressed proteins in imperatorin-treated KYSE150 cells (fold change ≥ 1.5).

**Accession ID**	**Gene symbol**	**Ratio H/L normalized**	**Protein name**
P04264	KRT1	7.3021	Keratin, type II cytoskeletal 1
Q9Y4C8	RBM19	6.6444	Probable RNA-binding protein 19
Q96EP0	RNF31	5.6044	E3 ubiquitin-protein ligase RNF31

Q96Q89	KIF20B	4.5026	Kinesin-like protein KIF20B
Q9Y294	ASF1A	3.2637	Histone chaperone ASF1A
Q9H857	NT5DC2	3.2266	5'-nucleotidase domain-containing protein 2
O15460	P4HA2	3.1937	Prolyl 4-hydroxylase subunit alpha-2 precursor
Q01995	TAGLN	3.1733	Transgelin
Q9NVX0	HAUS2	3.1413	HAUS augmin-like complex subunit 2
P50583	NUDT2	3.0568	Bis(5'-nucleosyl)-tetraphosphatase [asymmetrical]
Q9UBS8	RNF14	2.5776	E3 ubiquitin-protein ligase RNF14
Q5T0F9	CC2D1B	2.5328	Coiled-coil and C2 domain-containing protein 1B
Q9GZY4	COA1	2.454	Cytochrome c oxidase assembly factor 1 homolog
Q86VZ5	SGMS1	2.3823	Phosphatidylcholine:ceramide cholinephosphotransferase 1
P13796	LCP1	2.3185	Plastin-2
P07199	CENPB	2.2159	Major centromere autoantigen B
P22676	CALB2	2.1354	Calretinin
Q86WJ1	CHD1L	2.0078	Chromodomain-helicase-DNA-binding protein 1-like
Q96MF7	NSMCE2	2.0008	E3 SUMO-protein ligase NSE2
P05091	ALDH2	1.9745	Aldehyde dehydrogenase, mitochondrial precursor
Q93074	MED12	1.969	Mediator of RNA polymerase II transcription subunit 12
P57729	RAB38	1.9468	Ras-related protein Rab-38
Q96HY6	DDRGK1	1.942	DDRGK domain-containing protein 1 precursor
Q5PRF9	SAMD4B	1.9132	Protein Smaug homolog 2
P31321	PRKAR1B	1.9063	cAMP-dependent protein kinase type I-beta regulatory subunit
Q9Y3M8	STARD13	1.8967	StAR-related lipid transfer protein 13
Q04695	KRT17	1.8944	Keratin, type I cytoskeletal 17
Q6UX07	DHRS13	1.8912	Dehydrogenase/reductase SDR family member 13 precursor
P49207	RPL34	1.8738	60S ribosomal protein L34
Q6BDS2	UHRF1BP1	1.8406	UHRF1-binding protein 1
O15357	INPPL1	1.8262	Phosphatidylinositol 3,4,5-trisphosphate 5-phosphatase 2
P43121	MCAM	1.8246	Cell surface glycoprotein MUC18 precursor
Q5T653	MRPL2	1.7903	39S ribosomal protein L2, mitochondrial precursor
Q8TD19	NEK9	1.788	Serine/threonine-protein kinase Nek9
P51692	STAT5B	1.7596	Signal transducer and activator of transcription 5B
Q96QU8	XPO6	1.7532	Exportin-6
Q15904	ATP6AP1	1.7509	V-type proton ATPase subunit S1 precursor
Q9Y619	SLC25A15	1.7502	Mitochondrial ornithine transporter 1
P61225	RAP2B	1.7462	Ras-related protein Rap-2b precursor
Q9UKB1	FBXW11	1.7423	F-box/WD repeat-containing protein 11

P52566	ARHGDIB	1.7355	Rho GDP-dissociation inhibitor 2
Q8N8N7	PTGR2	1.7106	Prostaglandin reductase 2
Q9NP61	ARFGAP3	1.6811	ADP-ribosylation factor GTPase-activating protein 3
Q9BZQ8	FAM129A	1.6804	Protein Niban
Q14680	MELK	1.6484	Maternal embryonic leucine zipper kinase
Q5ZPR3	CD276	1.6451	CD276 antigen precursor
P04798	CYP1A1	1.6377	Cytochrome P450 1A1
P26038	MSN	1.6364	Moesin
Q9Y221	NIP7	1.6188	60S ribosome subunit biogenesis protein NIP7 homolog
Q5W0V3	FAM160B1	1.5918	Protein FAM160B1
Q9UBW7	ZMYM2	1.5863	Zinc finger MYM-type protein 2
P78540	ARG2	1.5843	Arginase-2, mitochondrial precursor
Q9UPQ0	LIMCH1	1.5791	LIM and calponin homology domains-containing protein 1
Q9GZV1	ANKRD2	1.5791	Ankyrin repeat domain-containing protein 2
Q9BYG5	PARD6B	1.5767	Partitioning defective 6 homolog beta
Q9NYY8	FASTKD2	1.5756	FAST kinase domain-containing protein 2, mitochondrial precursor
Q9NZQ7	CD274	1.5698	Programmed cell death 1 ligand 1 precursor
Q53HC9	TSSC1	1.5567	Protein TSSC1
Q9UP83	COG5	1.5529	Conserved oligomeric Golgi complex subunit 5
O96011	PEX11B	1.547	Peroxisomal membrane protein 11B
Q96MG7	NSMCE3	1.529	Non-structural maintenance of chromosomes element 3 homolog
P46100	ATRX	1.523	Transcriptional regulator ATRX
P00558	PGK1	1.5157	Phosphoglycerate kinase 1
O43819	SCO2	1.5114	Protein SCO2 homolog, mitochondrial precursor
Q08431	MFGE8	1.5061	Lactadherin precursor
Q9HD26	GOPC	1.5035	Golgi-associated PDZ and coiled-coil motif-containing protein
P34949	MPI	0.666	Mannose-6-phosphate isomerase
P16401	HIST1H1B	0.66431	Histone H1.5
Q09028	RBBP4	0.66359	Histone-binding protein RBBP4
Q86X29	LSR	0.66155	Lipolysis-stimulated lipoprotein receptor
Q13501	SQSTM1	0.66119	Sequestosome-1
O95684	FGFR1OP	0.66071	FGFR1 oncogene partner
P27701	CD82	0.65852	CD82 antigen
Q9Y5P4	COL4A3BP	0.65826	Collagen type IV alpha-3-binding protein
Q96HP4	OXNAD1	0.65452	Oxidoreductase NAD-binding domain-containing protein 1 precursor
Q14240	EIF4A2	0.6531	Eukaryotic initiation factor 4A-II
Q6KB66	KRT80	0.65195	Keratin, type II cytoskeletal 80

P83111	LACTB	0.65134	Serine beta-lactamase-like protein LACTB, mitochondrial precursor
P51636	CAV2	0.64302	Caveolin-2
Q9HAF1	MEAF6	0.64134	Chromatin modification-related protein MEAF6
Q8ND04	SMG8	0.63957	Protein SMG8
Q7Z739	YTHDF3	0.63668	YTH domain-containing family protein 3
Q99985	SEMA3C	0.63426	Semaphorin-3C precursor
Q01581	HMGCS1	0.63373	Hydroxymethylglutaryl-CoA synthase, cytoplasmic
Q14674	ESPL1	0.63357	Separin
Q16270	IGFBP7	0.62945	Insulin-like growth factor-binding protein 7 precursor
Q5HYK7	SH3D19	0.62388	SH3 domain-containing protein 19
Q96JQ2	CLMN	0.61893	Calmin
Q96SZ6	CDK5RAP1	0.61361	CDK5 regulatory subunit-associated protein 1
O94907	DKK1	0.60637	Dickkopf-related protein 1 precursor
Q9HBM1	SPC25	0.60353	Kinetochore protein Spc25
Q5VSL9	STRIP1	0.6035	Striatin-interacting protein 1
Q9H0W8	SMG9	0.59823	Protein SMG9
P59780	AP3S2	0.58408	AP-3 complex subunit sigma-2
Q96EK5	KIF1BP	0.58358	KIF1-binding protein
P31431	SDC4	0.58103	Syndecan-4 precursor
Q9H3L0	MMADHC	0.57773	Methylmalonic aciduria and homocystinuria type D protein, mitochondrial precursor
Q9H1Z4	WDR13	0.57405	WD repeat-containing protein 13
O95772	STARD3NL	0.56576	MLN64 N-terminal domain homolog
Q8N726	CDKN2A	0.55941	Tumor suppressor ARF
Q9BV57	ADI1	0.55927	1,2-dihydroxy-3-keto-5-methylthiopentene dioxygenase
Q8N9N8	EIF1AD	0.5577	Probable RNA-binding protein EIF1AD
Q9H5Z1	DHX35	0.54434	Probable ATP-dependent RNA helicase DHX35
P12277	CKB	0.53405	Creatine kinase B-type
Q6ZNJ1	NBEAL2	0.52321	Neurobeachin-like protein 2
Q9ULU4	ZMYND8	0.5226	Protein kinase C-binding protein 1
O75592	MYCBP2	0.52145	E3 ubiquitin-protein ligase MYCBP2
P07996	THBS1	0.51167	Thrombospondin-1 precursor
P52943	CRIP2	0.4989	Cysteine-rich protein 2
P13726	F3	0.48911	Tissue factor precursor
O43353	RIPK2	0.48776	Receptor-interacting serine/threonine-protein kinase 2
P61812	TGFB2	0.48612	Transforming growth factor beta-2 precursor
P05187	ALPP	0.48135	Alkaline phosphatase, placental type precursor
Q9Y2K1	ZBTB1	0.47934	Zinc finger and BTB domain-containing protein 1
Q9UBF8	PI4KB	0.45931	Phosphatidylinositol 4-kinase beta
P35527	KRT9	0.44911	Keratin, type I cytoskeletal 9
Q92545	TMEM131	0.44355	Transmembrane protein 131

P00742	F10	0.43054	Coagulation factor X precursor
Q86U90	YRDC	0.42717	YrdC domain-containing protein, mitochondrial precursor
Q13111	CHAF1A	0.40921	Chromatin assembly factor 1 subunit A
Q9H2P9	DPH5	0.35902	Diphthine methyl ester synthase
Q9Y3B1	PRELID3B	0.32672	PRELI domain containing protein 3B
Q8NCM8	DYNC2H1	0.24505	Cytoplasmic dynein 2 heavy chain 1
Q9P270	SLAIN2	0.22831	SLAIN motif-containing protein 2
Q14520	HABP2	0.22658	Hyaluronan-binding protein 2 precursor
O00391	QSOX1	0.20889	Sulfhydryl oxidase 1 precursor
P13645	KRT10	0.20158	Keratin, type I cytoskeletal 10

Supplementary Table 4. Correlation between p-CREB1 expression level and clinicopathological parameters in 186 cases of esophageal cancer.

Variable	n	Low p-CREB1	High p-CREB1	*P* value [a]
Age (years)				
≤55	29	19	10	
>55	157	100	57	0.850
Gender				
Male	144	88	56	
Female	42	31	11	0.131
T-Stage				
1/2	35	26	9	
3/4	141	86	55	0.143
N-Stage				
N0	86	63	23	
N1	100	56	44	**0.014***
M-Stage				
M0	186	119	67	
M1	0	0	0	1
Grade				
I & II	144	89	55	
III & IV	42	30	12	0.253

[a] Pearson Chi-square. Statistical significance (P <0.05) is shown in bold.

Supplementary Table 5. The sequences of TGFβ2 siRNA and CREB1 sgRNA.

Name		Sequence
TGFβ2 siRNA	TGFβ2 #1	GCAATGGAGAAGAATGCTTAAGCATTCTTCTCCATTGC
	TGFβ2 #2	GGATTGAGCTATATCAGATATCTGATATAGCTCAATCC
CREB1 sgRNA	CREB1 #1	GCCGCTGCGCACTCGGCACT
	CREB1 #2	ACAGATTGCCACATTAGCCC

Supplementary Table 6. The list of primers used for gene cloning, qPCR and mutation.

Primer Name		Primer sequence
CREB1 subcloning	Forward	5'- AGTGGATCCATGACCATGGAATCTGGAGCCGAGAAC-3'
	Reverse	5'- CCGGAATTCATCTGATTTGTGGCAGTAAAGGTCCT-3'
TGFβ2 promoter cloning	Forward	5'- CCGCTCGAGATCTTCTCTCAGTAGCTCTGGTTTG-3'
	Reverse	5'- CCCAAGCTTGCTGCCAGCAGATAACATCA-3'
TGFβ2 qPCR	Forward	5'- GAGGGATCTAGGGTGGAA-3'
	Reverse	5'- GCTGTGCTGAGTGTCTGAA-3'
CCL2 qPCR	Forward	5'- CAGCCAGATGCAATCAATGCC-3'
	Reverse	5'- TGGAATCCTGAACCCACTTCT-3'
TGFβ2 promoter mutation#1	Forward	5'-CTGACTGTAATCCTAGCATGTCACTTTGTTG-3'
	Reverse	5'-ATGCTAGGATTACAGTCAGAAGTCCTCTCG-3'
TGFβ2 promoter mutation#2	Forward	5'-GACTGTAATCCTAGCACGGCACTTTGTTGAAG-3
	Reverse	5'-CCGTGCTAGGATTACAGTCAGAAGTCCTCT-3'
CREB1-K304E mutation	Forward	5'- CGAGAGTGTCGTAGAAAGGAGAAAGAATAT -3'
	Reverse	5'- CCTTTCTACGACACTCTCGAGCTGCTTCCCT-3'
CREB1-K305E mutation	Forward	5'-GAGTGTCGTAGAAAGAAGGAAGAATATGTG-3'
	Reverse	5'-cCTTCTTTCTACGACACTCTCGAGCTGCTTC-3'
CREB1-K309E mutation	Forward	5'- AAGAAGAAAGAATATGTGGAATGTTTAGAA-3'
	Reverse	5'- cCACATATTCTTTCTTCTTTCTACGACACTC-3'
